# Multiple kernel learning for integrative consensus clustering of omic datasets

**DOI:** 10.1093/bioinformatics/btaa593

**Published:** 2020-06-27

**Authors:** Alessandra Cabassi, Paul D W Kirk

**Affiliations:** MRC Biostatistics Unit, University of Cambridge, Cambridge CB2 0SR, UK; MRC Biostatistics Unit, University of Cambridge, Cambridge CB2 0SR, UK; Cambridge Institute of Therapeutic Immunology & Infectious Disease, University of Cambridge, Cambridge CB2 0AW, UK

## Abstract

**Motivation:**

Diverse applications—particularly in tumour subtyping—have demonstrated the importance of integrative clustering techniques for combining information from multiple data sources. Cluster Of Clusters Analysis (COCA) is one such approach that has been widely applied in the context of tumour subtyping. However, the properties of COCA have never been systematically explored, and its robustness to the inclusion of noisy datasets is unclear.

**Results:**

We rigorously benchmark COCA, and present Kernel Learning Integrative Clustering (KLIC) as an alternative strategy. KLIC frames the challenge of combining clustering structures as a multiple kernel learning problem, in which different datasets each provide a *weighted* contribution to the final clustering. This allows the contribution of noisy datasets to be down-weighted relative to more informative datasets. We compare the performances of KLIC and COCA in a variety of situations through simulation studies. We also present the output of KLIC and COCA in real data applications to cancer subtyping and transcriptional module discovery.

**Availability and implementation:**

R packages *klic* and *coca* are available on the Comprehensive R Archive Network.

**Supplementary information:**

Supplementary data are available at *Bioinformatics* online.

## 1 Introduction

Thanks to technological advances, both the availability and the diversity of omic datasets have hugely increased in recent years (Manzoni *et al.*, 2018). These datasets provide information on multiple levels of biological systems, going from the genomic and epigenomic level, to gene and protein expression level, up to the metabolomic level, accompanied by phenotype information. Many publications have highlighted the importance of integrating information from diverse omic datasets in order to provide novel biomedical insight. For example, numerous studies by The Cancer Genome Atlas (TCGA) consortium have demonstrated the value of combining multiple omic datasets in order to define cancer subtypes (see e.g. The Cancer Genome Atlas Research Network, 2011, 2012).

Many existing statistical and computational tools have been applied to this problem and many others have been developed specifically for this. One of the first statistical methods applied to integrative clustering for cancer subtypes was *iCluster* (Shen *et al.*, 2009, 2013). iCluster finds a partitioning of the tumours into different subtypes by projecting the available datasets onto a common latent space, maximizing the correlation between data types. Another statistical method for integrative clustering is *Multiple Dataset Integration* (MDI; see Kirk *et al.*, 2012; Mason *et al.*, 2016). It is based on Dirichlet-multinomial mixture models in which the allocation of observations to clusters in one dataset influences the allocation of observations in another, while allowing different datasets to have different numbers of clusters. Similarly, *Bayesian Consensus Clustering* (BCC) is based on a Dirichlet mixture model that assigns a different probability model to each dataset. Again, samples belong to different partitions, each given by a different data type, but here they also adhere loosely to an overall clustering (Lock and Dunson, 2013). More recently, Gabasová *et al.* (2017b) developed *Clusternomics*, a mixture model over all possible combinations of cluster assignments on the level of individual datasets that allows to model different degrees of dependence between clusters across datasets.

Integrative clustering methods can be broadly classified as either *joint modelling* or *two-step* approaches. The former simultaneously consider all datasets together (e.g. MDI or BCC). The latter, which we consider here, are composed of two steps: first, the clustering structure in each dataset is analyzed independently; then an integration step is performed to find a common clustering structure that combines the individual ones. These approaches have sometimes also been referred to as *sequential analysis* methods (Kristensen *et al.*, 2014).

Cluster Of Clusters Analysis (COCA) is a particular two-step approach, which has grown in popularity since its first introduction in The Cancer Genome Atlas Research Network (2012). As we explain in Section 2.1, COCA proceeds by first clustering each of the datasets separately, and then building a binary matrix that encodes the cluster allocations of each observation in each dataset. This binary matrix is then used as the input to a CC algorithm (Monti *et al.*, 2003; Wilkerson and Hayes, 2010), which returns a single, global clustering structure, together with an assessment of its stability. The idea is that this global clustering structure both combines and summarizes the clustering structures of the individual datasets. Despite its widespread use, to the best of our knowledge the COCA algorithm has never previously been systematically explored. In what follows, we elucidate the algorithm underlying COCA, and highlight some of its limitations. We show that one key limitation is that the combination of the clustering structures from each dataset is *unweighted*, making the output of the algorithm sensitive to the inclusion of poor quality datasets.

An alternative class of approaches for integrating multiple omic datasets is provided by those based on *kernel methods* (see, among others, Lanckriet *et al.*, 2004b; Lewis *et al.*, 2006, for ‘omic dataset’ applications). In these, a kernel function (which defines similarities between different units of observation) is associated with each dataset. These may be straightforwardly combined in order to define an overall similarity between different units of observation, which incorporates similarity information from each dataset. Determining an optimal (weighted) combination of kernels is known as *multiple kernel learning* (MKL); see, e.g. Bach *et al.* (2004), Gönen and Alpayd (2011), Lanckriet *et al.* (2004a), Strauß *et al.* (2019), Wang *et al.* (2017), Yu *et al.* (2010). A challenge associated with these approaches is how best to define the kernel function(s), for which there may be many choices.

Here, we combine ideas from COCA and MKL in order to propose a new Kernel Learning Integrative Clustering (KLIC) method that addresses the limitations of COCA (Section 2.2). Key to our approach is the result that the *consensus matrix* returned by CC is a valid kernel matrix (Section 2.2.3). This insight allows us to make use of the full range of MKL approaches in order to combine consensus matrices derived from different omic datasets. We perform simulation studies to illustrate our proposed approach and compare it with COCA. Finally, we show how KLIC and COCA compare in two practical applications: multiplatform tumour subtyping, where the goal is to stratify patients, and transcriptional module discovery, where genes are the statistical observations that we want to cluster.

## 2 Materials and methods

### 2.1 Cluster of clusters analysis

*COCA* (The Cancer Genome Atlas Research Network, 2012) is an integrative clustering method that was first introduced in a breast cancer study by The Cancer Genome Atlas Research Network (2012) and quickly became a popular tool in cancer studies (see e.g. Aure *et al.*, 2017; Hoadley *et al.*, 2014). It makes use of *CC* (Monti *et al.*, 2003), an algorithm that was originally developed to assess the stability of the clusters obtained with any clustering algorithm.

#### 2.1.1 Consensus clustering

We recall here the main features of CC in order to be able to explain the functioning of COCA. As originally formulated, CC is an approach for assessing the robustness of the clustering structure present in a single dataset (Monti *et al.*, 2003; Wilkerson and Hayes, 2010). The idea behind CC is that, by re-sampling multiple times the items that we want to cluster and then applying the same clustering algorithm to each of the subsets of items, we assess the robustness of the clustering structure that the algorithm detects, both to perturbations of the data and (where relevant) to the stochasticity of the clustering algorithm. To do this, CC makes use of the concepts of co-clustering matrix and consensus matrix, which we recall here.

Given a set of items $X=[x_{1},\ldots ,x_{N}]$ that we seek to cluster and a clustering $c=[c_{1},\ldots ,c_{N}]$ such that *c_i_* is the label of the cluster to which item $x_{i}$ has been assigned, the corresponding *co-clustering matrix* (or *connectivity matrix*) is an *N *×* N* matrix *C* such that the *ij*th element *C_ij_* is equal to one if *c_i_* = *c_j_*, and zero otherwise. Let $X^{\left(1\right)},\ldots ,X^{\left(H\right)}$ be a list of perturbed datasets obtained by re-sampling subsets of items and/or covariates from the original dataset *X*. If $I^{\left(h\right)}$ is the subset of the indices of the observations I={1,2,…,N} present in $X^{\left(h\right)}$, then the co-clustering matrix has *ij*-th element equal to one if $i,j\in I^{\left(h\right)}$ and *c_i_* = *c_j_*, zero otherwise. We denote by $C^{\left(h\right)}$ the co-clustering matrix corresponding to dataset $X^{\left(h\right)}$ where the items have been assigned to *K* classes using a clustering algorithm.

The *consensus matrix* $\Delta^{K}$ is an *N *×* N* matrix with elements

(1) $$\Delta_{ij}^{K}=\frac{\sum_{h=1}^{H}C_{ij}^{\left(h\right)}}{\min\left\{1,\sum_{h=1}^{H}I_{ij}^{\left(h\right)}\right\}}$$

where $I_{ij}^{\left(h\right)}=1$ if both items *i* and *j* are present in dataset $X^{\left(h\right)}$.

Thus, CC performs multiple runs of a (stochastic) clustering algorithm (e.g. *k*-means, hierarchical clustering etc.) to assess the stability of the discovered clusters, with the consensus matrix providing a convenient summary of the CC analysis. If all the elements of the consensus matrix are close to either one or zero, this means that every pair of items is either almost always assigned to the same cluster, or almost always assigned to different clusters. Therefore, consensus matrices with all the elements close to either zero or one indicate stable clusters. In the framework of CC, these matrices can also be used to determine the number of clusters, by computing and comparing the consensus matrices $\Delta^{K}$ for a range of numbers of clusters $K=\{K_{\min},\ldots ,K_{\max}\}$ of interest and then pick the value of *K* that gives the consensus matrix with the greater proportion of elements close to either zero or one (Monti *et al.*, 2003).

#### 2.1.2 Cluster Of Clusters Analysis

In contrast to CC (which we emphasize is concerned with assessing clustering stability when analyzing a single dataset), the main goal of COCA is to summarize the clusterings found in *different* omic datasets, by identifying a ‘global’ clustering across the datasets that is intended to summarize the clustering structures identified in each of the individual datasets. In the first step, a clustering $c^{m}$ is produced independently for each dataset *X_m_*, m=1,…,M, each with a different number of clusters *K_m_*. We define $\bar{K}=\sum_{m=1}^{M}K_{m}$. Then, the clusters are summarized into a Matrix Of Clusters (MOC) of size $\bar{K}\times N$, with elements:

(2) $${\text{MOC}}_{n,m_{k}}=\{\begin{array}{cc} 1 & \text{if}\;c_{n}^{m}=m_{k},\\ 0 & \text{otherwise}. \end{array}$$

where by *m_k_* we denote the *k*th cluster in dataset *m*, $k=1,\ldots ,K_{m}$ and m=1,…,M. The MOC matrix is then used as input to CC together with a fixed global number of clusters *K*. The resulting consensus matrix is then used as the similarity matrix for a hierarchical clustering method (or any other distance-based clustering algorithm).

The global number of clusters *K* is not always known. In The Cancer Genome Atlas Research Network (2012), where COCA was introduced, the global number of clusters was chosen as in Monti *et al.* (2003), as explained above: CC was performed with different values of *K* and then the one that gave the ‘best’ consensus matrices were considered. Instead, Aure *et al.* (2017) suggest to choose the value of *K* that maximizes the average silhouette (Rousseeuw, 1987) of the final clustering, since this was found to give more sensible results.

Since the construction of the MOC matrix just requires the cluster allocations, COCA has the advantage of allowing clusterings derived from different sources to be combined, even if the original datasets are unavailable or unwieldy. However, this method is unweighted, since all the clusters found in the first step have the same influence on the final clustering. Moreover, the objective function that is optimized by the algorithm is unclear.

In what follows, we describe an alternative way of performing integrative clustering that takes into account not only the clusterings in each dataset, but also the information about the similarities between items that are extracted from different types of data. Additionally, the new method allows weights to be given to each source of information, according to how useful it is for defining the final clustering.

### 2.2 Kernel learning integrative clustering

Before introducing the new methodology, we recall the main principles behind the methods that we use to combine similarity matrices.

#### 2.2.1 Kernel methods

Using kernel methods, it is possible to model non-linear relationships between the data points with a low computational complexity, thanks to the so-called *kernel trick.* For this reason, these have been widely used to extend many traditional algorithms to the non-linear framework, such as PCA (Schölkopf *et al.*, 1998), linear discriminant analysis (Baudat and Anouar, 2000; Mika *et al.*, 1999; Roth and Steinhage, 2000) and ridge regression (Friedman *et al.*, 2001; Shawe-Taylor and Cristianini, 2004).

A *positive definite kernel* or, more simply, a *kernel δ* is a symmetric map δ:X×X→R for which for all $x_{1},x_{2},\ldots ,x_{N}\in X$, the matrix Δ with entries $\Delta_{ij}=\delta (x_{i},x_{j})$ is positive semi-definite. The matrix Δ is called the *kernel matrix* or *Gram matrix*. Kernel methods proceed by embedding the observations into a higher-dimensional feature space H endowed with an inner product ${\langle \cdot ,\cdot \rangle }_{H}$ and induced norm $\|\cdot {\|}_{H}$, making use of a map ϕ:X→H. Using Mercer’s theorem, it can be shown that for any positive semi-definite kernel function, *δ*, there exists a corresponding feature map, ϕ:X→H (see e.g. Vapnik, 1998). That is, for each kernel *δ*, there exists a feature map ϕ taking value in some inner product space H such that $\delta (x,x\prime )={\langle \phi (x),\phi (x\prime )\rangle }_{H}$. In practice, it is therefore often sufficient to specify a positive semi-definite kernel matrix, Δ, in order to allow us to apply kernel methods such as those presented in the following sections. For a more detailed discussion of kernel methods, see e.g. Shawe-Taylor and Cristianini (2004).

#### 2.2.2 Localized multiple kernel *k*-means clustering

Kernel *k*-means is a generalization of the *k*-means algorithm of Steinhaus (1956) to the kernel framework (Girolami, 2002). The kernel trick is used to reformulate the problem of minimizing the sum of squared distances between each point and the corresponding cluster centre (in the feature space) as a trace maximization problem that only requires knowing the Gram matrix corresponding to the kernel of interest. Optimal cluster allocations can then be efficiently determined using kernel Principal Component Analysis (PCA) . More details on kernel *k*-means can be found in the Supplementary Material.

The clustering algorithm used here is the extension of the kernel *k*-means approach to MKL (Gönen and Alpayd, 2011) with sample-specific weights (Gönen and Margolin, 2014) aimed at removing sample-specific noise. We consider multiple datasets $X_{1},\ldots ,X_{M}$ each with a different mapping function $\phi_{m}:R^{P}\to H_{m}$ and corresponding kernel $\delta_{m}(x_{i},x_{j})={\langle \phi_{m}(x_{i}),\phi_{m}(x_{j})\rangle }_{H_{m}}$ and kernel matrix Δ_*m*_. Then, if we define $\phi_{\Theta }(x_{i})=[\theta_{i1}\phi_{1}(x_{i})\prime ,\theta_{i2}\phi_{2}(x_{i})\prime ,\ldots ,\theta_{iM}\phi_{M}(x_{i})\prime ]\prime$, where $\Theta \in R_{+}^{N\times M}$ is a vector of kernel weights with elements *θ_im_* such that $\sum_{m}\theta_{im}=1$ and $\theta_{im}\geq 0$ for i=1,…,N, the kernel function of this multiple feature problem is a convex sum of the single kernels:

(3) $$\delta_{\Theta }(x_{i},x_{j})={\langle \phi_{\Theta }(x_{i}),\phi_{\Theta }(x_{j})\rangle }_{H_{m}}=\sum_{m=1}^{M}\theta_{im}\theta_{jm}\delta_{m}(x_{i},x_{j}).$$

We denote the corresponding kernel matrix by $\Delta_{\Theta }$. The idea of localized multiple kernel *k*-means is to replace the Gram matrix used in kernel *k*-means by this weighted matrix. The optimization strategy proposed by Gönen and Margolin (2014) is based on the idea that, for some fixed vector of weights Θ, this is a standard kernel *k*-means problem. Therefore, they develop a two-step optimization strategy: (i) given a fixed vector of weights Θ, solve the optimization problem as in the case of one kernel, with kernel matrix given by $\delta_{\Theta }$ and then (ii) minimize the objective function with respect to the kernel weights, keeping the assignment variables fixed. This is a convex quadratic programming (QP) problem that can be solved with any standard QP solver up to a moderate number of kernels *M*.

#### 2.2.3 Identifying consensus matrices as kernels

We prove that the consensus matrices defined in Section 2.1 are positive semi-definite, and hence that they can be used as input for any kernel-based clustering method, including the integrative clustering method presented in the next section. Given any *N *×* N* co-clustering matrix *C*, we can reorder the rows and columns to obtain a block-diagonal matrix with blocks $J_{1},J_{2},\ldots ,J_{K}$where *K* is the total number of clusters and *J_k_* is an $n_{k}\times n_{k}$ matrix of ones, with *n_k_* being the number of items in cluster *k*. It is straightforward to show that the eigenvalues of a block-diagonal matrix are simply the eigenvalues of its blocks. Since each block is a matrix of ones, the eigenvalues of each block are nonnegative, and so any co-clustering matrix *C* is positive semi-definite. Moreover, given any set of *λ_m_*, m=1,…,M non-negative, and co-clustering matrices *C_m_*, m=1,…,M, then $\sum_{m=1}^{M}\lambda_{m}C_{m}$ is positive semi-definite, because if *λ* is a nonnegative scalar, and *C* is positive semi-definite, then *λC* is also positive semi-definite and the sum of positive semi-definite matrices is a positive semi-definite matrix. Since every consensus matrix is of the form $\sum_{m}\lambda_{m}C_{m}$, we can conclude that any consensus matrix is positive semi-definite.

#### 2.2.4 Kernel Learning Integrative Clustering

We recall from Section 2.2.1 that any positive semi-definite matrix defines a feature map $\phi :R^{P}\to H$ and is therefore a valid kernel matrix. The integrative clustering method that we introduce here is based on the idea that we can identify the consensus matrices produced by CC as kernels. That is, one can perform CC on each dataset to produce a consensus matrix Δ_*m*_ for each m∈{1,…,M}. This is a kernel $\Delta_{m}$, where the *ij*th element corresponds to the similarity between items *i* and *j*. Therefore, these matrices Δ_*m*_ can be combined through the (localized) multiple kernel *k*-means algorithm described in Section 2.2.2. This allows a weight to be obtained for each kernel, as well as a global clustering ***c*** of the items. We note that this algorithm could also be applied using more than one similarity matrix per dataset, and also using kernel matrices other than (or in addition to) consensus matrices.

## 3 Examples

### 3.1 Simulated data

To assess the KLIC algorithm described in Section 2.2.4 and to compare it with COCA, we perform a range of simulation studies. We generate several synthetic datasets, each composed of data belonging to six different clusters of equal size. Each dataset has total number of observations equal to 300. Each observation $x_{n}^{\left(k\right)}$ is generated from a bivariate normal with mean *ks* for each variable, where *k* denotes the cluster to which the observation belongs and *s* the separation level of the dataset. Higher values of *s* give clearer clustering structures. The variance covariance matrix is the identity matrix.

**Fig. 1.**
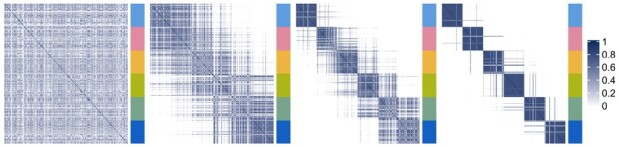
Consensus matrices of the synthetic data with different levels of noise, going from ‘no cluster separability’ to ‘high cluster separability’. Blue indicates high similarity. The colours of the bar to the right of each matrix indicate the cluster labels

We consider the following settings:

1. *Similar datasets.* We generate four datasets that have the same clustering structure and cluster separability *s*. We denote the datasets by A, B, C and D. The goal of this experiment is to show that using localized kernel *k*-means on multiple consensus matrices leads to better results than those obtained using just one consensus matrix. To demonstrate how we may deal with irrelevant variables, we also repeat this experiment adding to each dataset 13 variables centred at zero that have no clustering structure, i.e.
  (4) $$x_{1}^{\left(k\right)},\ldots ,x_{50}^{\left(k\right)}∼N([ks,ks,\underset{13}{\underset{︸}{0,\ldots ,0}}],I),\;\forall k=1,\ldots ,6,$$
  where *I* is the 15 × 15 identity matrix.
2. *Datasets with different levels of noise.* In this case we utilize four datasets that have the same clustering structure, but different levels of cluster separability *s*. We denote the datasets by 0 for ‘no cluster separability’, 1 ‘low cluster separability’, 2 ‘medium cluster separability’ and 3 ‘high cluster separability’ (Fig. 1). We use this example to show how the weights are allocated to each consensus matrix and why it is important to assign lower weights to datasets that are noisy or not relevant.

We repeat each experiment 100 times. For each synthetic dataset, we use CC to obtain the consensus matrices. For simplicity, we set *K *=* *6. As for the clustering algorithm, we use *k*-means clustering with Euclidean distance, which we found to work well in practice. The Supplementary Material contains additional simulation settings. In particular, we consider a wide range of separability values for the setting with four similar datasets and the integration of datasets with nested clusters. Moreover, we perform a short sensitivity analysis of the choice or tuning options for the *k*-means algorithm.

### 3.2 Multiplatform analysis of 12 cancer types

Hoadley *et al.* (2014) performed a multiplatform integrative analysis of 3,527 tumour samples from 12 different tumour types, and used COCA to identify 11 integrated tumour subtypes. To do so, they applied different clustering algorithms to each data type separately: DNA copy number, DNA methylation, mRNA expression, microRNA expression and protein expression. They then combined the five sets of clusters obtained in this way using COCA. The final clusters are highly correlated with the tissue-of-origin of each tumour sample, but some cancer types coalesce into the same clusters. The clusters obtained in this way were shown to be prognostic and to give independent information from the tissue-of-origin.

Here, we use the same data to try to replicate their analysis, and compare the clusters obtained with COCA to those obtained with KLIC. To facilitate future analyses by other researchers, we have made available our scripts for processing and analyzing these datasets using the freely available R statistical programming language (R Core Team, 2020), which include scripts that seek to replicate the original analysis of Hoadley *et al.* (2014), at https://github.com/acabassi/klic-pancancer-analysis.

### 3.3 Transcriptional module discovery

*Transcriptional modules* are groups (i.e. clusters) of genes that share a common biological function and are co-regulated by a common set of transcription factors. It has been recognized that integrative clustering methods can be useful for discovering transcriptional modules, by combining gene expression datasets with datasets that provide information about transcription factor binding (Ihmels *et al.*, 2002; Savage *et al.*, 2010).

Here, we consider transcriptional module discovery for yeast (*Saccharomyces cerevisiae*). We integrate the expression dataset of Granovskaia *et al.* (2010) that contains measurements related to 551 genes whose expression profiles have been measured at 41 different time points of the cell cycle with the ChIP-chip dataset of Harbison *et al.* (2004) which provides binding information for 117 transcriptional regulators for the same genes. The latter was discretized as in Kirk *et al.* (2012) and Savage *et al.* (2010).

## 4 Results

### 4.1 Simulated data

In Section 4.1, we apply the developed methods to the synthetic datasets. In Section 4.1.2, we compare the performances of our method for integrative clustering to COCA and other integrative clustering algorithms.

#### 4.1.1 Kernel Learning Integrative Clustering

We apply KLIC to the synthetic datasets presented in Section 3.1.

##### 4.1.1.1 Similar datasets

**Fig. 2.**
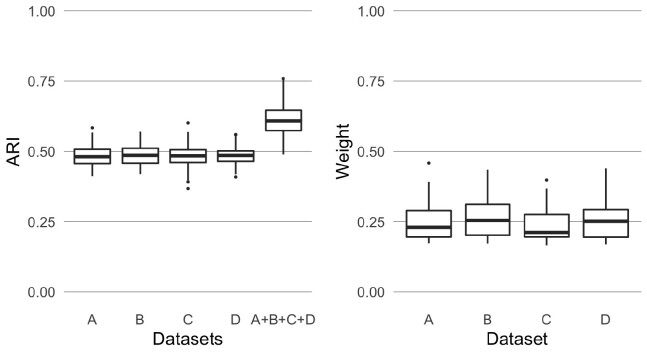
Results of applying KLIC to four similar datasets. Left: ARI of KLIC applied to each dataset separately (columns ‘A’, ‘B’, ‘C’ and ‘D’) and to all four datasets together (column ‘A + B+C + D’). The ARI is higher in the last column because KLIC can combine information from all the datasets to find a global clustering. Right: kernel weights associated to each dataset, when applying KLIC to all four datasets together. The algorithm is able to recognize that each dataset contains the same amount of information regarding the global clustering, and therefore assigns on average the same weight to each dataset

First, we run the kernel *k*-means algorithm on each of the consensus matrices that have the same clustering structure and noise level. To assess the quality of the clustering, we compare the clustering found with the true one using the adjusted Rand index (ARI; Rand, 1971), which is equal to one if they are equal and is equal to zero if we observe as many similarities between the two partitions of the data as it is expected by chance. Then we run KLIC on multiple datasets. In Figure 2, the box plots of the ARI obtained combining the four datasets together using KLIC (column ‘A + B+C + D’) and the box plots of the average weights assigned by the KLIC algorithm to the observations in each dataset are reported. We observe that as expected, combining together more datasets helps recovering the clustering structure better than just taking the matrices one at a time. This is because localized kernel *k*-means allows to give different weights to each observation. Therefore, if data point *n* is hard to classify in Dataset *d*_1_, but not in Dataset *d*_2,_ we will have $\theta_{nd_{1}}<\theta_{nd_{2}}$. However, on average the weights are divided equally between the datasets. This reflects the fact that all datasets have the same dispersion and, as a consequence, they contain on average the same amount of information about the clustering structure.

##### 4.1.1.2 Datasets with different levels of noise

**Fig. 3.**
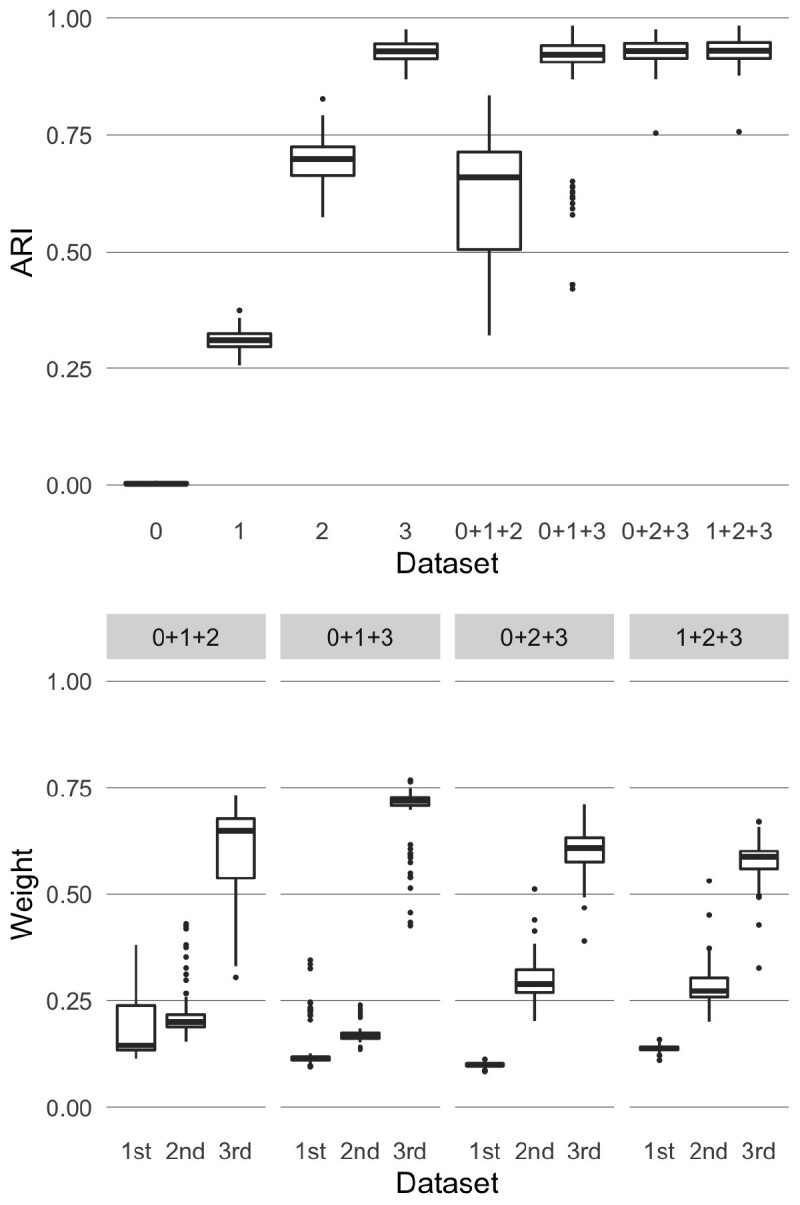
Results of applying KLIC to datasets with different levels of noise (‘0’ indicates the dataset that has no cluster separability, ‘1’ the dataset with low cluster separability, and so on). Top: ARI of KLIC applied to each dataset separately (columns ‘0’, ‘1’, ‘2’ and ‘3’) and to subsets of three of those datasets (columns ‘0 + 1 + 2’, ‘0 + 1 + 3’, ‘0 + 2 + 3’ and ‘1 + 2 + 3’). Bottom: kernel weights associated to each dataset in each of the experiments with multiple datasets, ordered by cluster separability. For example, the first subset is ‘0 + 1 + 2’ so the weights marked as ‘first’ are those assigned to dataset ‘0’, ‘second’ are those assigned to ‘1’ and so on. For each subset of datasets the weights of the noisier datasets (‘first’ and ‘second’) are lower than those of the ‘best’ dataset in the subset (‘third’). This is reflected in an increased ARI in each subset, compared with applying KLIC to those datasets separately

Here we use the datasets shown in Figure 1, that have the same clustering structure (six clusters of the same size each) but different levels of cluster separability. We consider four different settings, each time combining three out of the four synthetic datasets. Figure 3 shows the box plots of the ARI obtained using kernel *k*-means on the datasets taken one at a time (columns ‘0’, ‘1’, ‘2’, ‘3’) and the ARI obtained using KLIC on each subset of datasets (columns “0 + 1 + 2”, “0 + 1 + 3”, “0 + 2 + 3”, “1 + 2 + 3”). As expected, the consensus matrices with clearer clustering structure give higher values of the ARI on average. Moreover, the ARI obtained combining three matrices with different levels of cluster separability is on average the same or higher as in the case when only the “best” matrix is considered. This is because larger weights are assigned to the datasets that have clearer clustering structure. In the bottom part of Figure 3 are reported the box plots of the average weights given by the localized multiple kernel *k*-means to the observations in each dataset. It is easy to see that each time the matrix with best cluster separability has higher weights than the other two.

#### 4.1.2 Comparison between KLIC, COCA and other methods

We compare the performance of KLIC to the one obtained using COCA, as well as to two other comparable integrative clustering algorithms for which implementations are readily available; namely, iCluster and Clusternomics. Additionally, we compare with localized multiple kernel *k*-means using standard radial basis function (RBF) kernels. We use the same synthetic datasets as in the previous section.

For COCA, we use the *k*-means algorithm with Euclidean distance, fixing the number of clusters to be equal to the true one, to find the clustering labels of each dataset. Many other clustering algorithms can be used, but this is the one that gives the best results among the most common ones. To find the global clustering, we build the consensus matrices using 1000 re-samplings of the data, each time with 80% of the observations and all the features. The final clustering is done using hierarchical clustering with average linkage on the consensus matrix. The iCluster model is fitted using the tune.iCluster2 function of the R package *iCluster* (Shen, 2012), with number of clusters set to six. For Clusternomics we use the contextCluster function of the R package *clusternomics*  Gabasová (2017a), providing the true number of clusters both for the partial and global clusterings. To assess the impact of RBF kernel parameter choice, we consider two ways to set the free parameter, *σ*, of the kernel. In one setting we fix *σ *= 1, a common default value. In the second setting, *σ* is tuned for each dataset to maximize the average ARI between the clustering obtained with kernel *k*-means using the RBF kernel and the true clusters (more information about this procedure can be found in the Supplementary Material). Although this procedure clearly could not be applied in practice (where the true clustering is unknown), it is used here to determine a putative upper bound on the performances of MKL with this kernel.

##### 4.1.2.1 Similar datasets

**Fig. 4.**
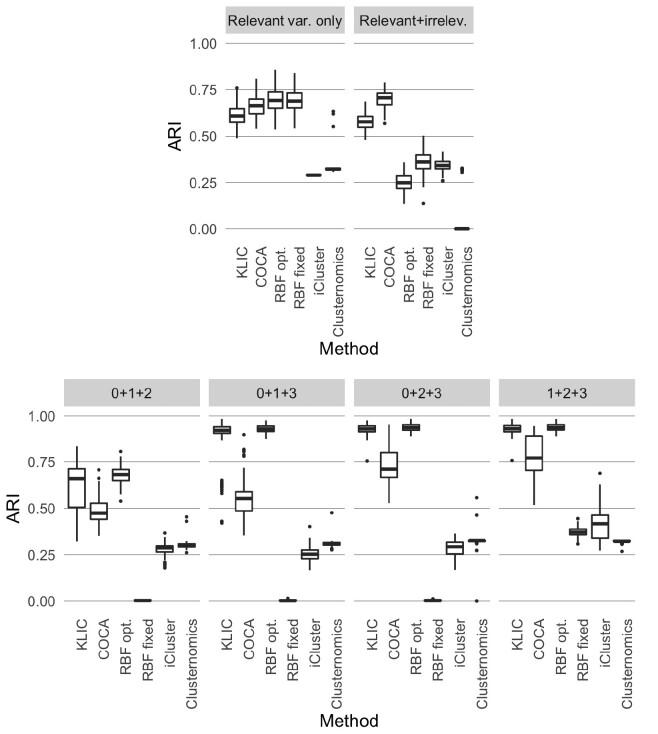
Comparison between KLIC, COCA and other clustering algorithms. The labels ‘RBF opt.’ and ‘RBF fixed’ refer to the MKL method using an RBF kernel with either *σ* optimized or fixed at 1 (see text). Top: ARI obtained with each clustering algorithm using four datasets having the same clustering structure and cluster separability (as in Fig. 2). Bottom: ARI obtained with COCA and KLIC for each of the subsets of heterogeneous datasets considered in Figure 3. The high ARI obtained with KLIC in all settings shows the advantage of using this method, especially when some of the datasets are noisy

We combine four datasets that have the same clustering structure and cluster separability. In Figure 4, the ARI of all considered methods applied to 100 sets of data of this type is shown. In the first setting, where only variables relevant for the clustering are present, the localized multiple kernel *k*-means with RBF kernel has the highest median ARI, followed by COCA and KLIC. To cluster the data that include noisy variables, we replace the *k*-means algorithm by the sparse *k*-means feature selection framework of Witten and Tibshirani (2010) in COCA and KLIC, using the R package *sparcl* (Witten and Tibshirani, 2018). Thanks to this, the performances of these two methods are not affected by the presence of irrelevant variables. COCA, in particular, has the highest median ARI, followed by KLIC. This shows that both methods work well in the case of multiple datasets that have the same clustering structure and level of noise and, in contrast to the four other methods considered here, can be straightforwardly modified to deal with the presence of irrelevant features.

##### 4.1.2.2 Datasets with different levels of noise

We also compare the behaviour of all methods in the presence of multiple datasets with the same clustering structure, but different levels of cluster separability. The ARI is shown in Figure 4. We observe that, in each of the four simulation settings, KLIC and the optimized version of localized multiple kernel *k*-means with RBF kernel have the highest ARI scores. The reason for this is that COCA, iCluster and Clusternomics are not weighted methods, so their ability to recover the true clustering structure is decreased by adding noisy datasets. Instead, we have shown in the previous section that KLIC allows to give lower weights to the noisiest datasets, achieving better performances. We emphasize that the optimal values of the RBF parameters have been determined making use of the true cluster labels, which will not be possible in real applications. The performance achieved when the RBF kernel parameter, *σ*, is fixed to 1 may therefore be more representative of what can be achieved in practice.

Overall, these comparisons suggest that KLIC may be a good default choice, since it can be run in such a way that it is robust to both the inclusion of noisy variables (via the choice of an appropriate clustering algorithm) and of noisy datasets.

### 4.2 Multiplatform analysis of 12 cancer types

**Fig. 5.**
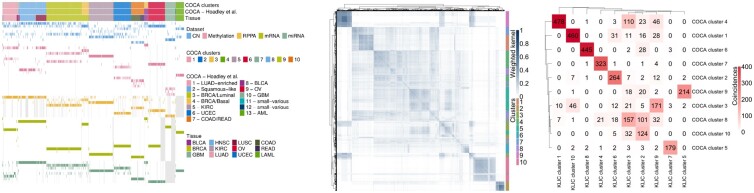
Multiplatform analysis of 12 cancer types. Left: matrix-of-clusters of the pan-cancer data, each row corresponds to a cluster in one of the dataset, and each column corresponds to a tumour sample. Coloured cells show which tumours belong to each cluster. Grey cells indicate missing observations. Centre: weighted similarity matrix. Right: Coincidence matrix comparing the clusters given by COCA and KLIC

The first step of the data analysis is dedicated to replicating the analysis performed by Hoadley *et al.* (2014). The DNA copy number, DNA methylation, mRNA expression, microRNA expression, and protein expression data were preprocessed in the same way as Hoadley *et al.* (2014) did. We then clustered the tumour samples independently for each dataset, using the same clustering algorithm as in the original paper. We compared the clusters we obtained to those reported by Hoadley *et al.* (2014) for different number of clusters, and we found that the best correspondence was given by choosing the same number of clusters as in the original paper, except for the microRNA expression data, for which we found the best number of clusters to be seven (instead of 15). Figure 5 (left) shows the MOC matrix formed by these clusters and the resulting COCA clusters. As can be seen from the Figure 5, each dataset has some missing observations. The corresponding entries in the MOC matrix were set to zero. We chose the number of clusters that maximizes the silhouette, as suggested by Aure *et al.* (2017), which is 10.

We then applied KLIC to the preprocessed data, building one consensus matrix for each dataset, using the same clustering algorithm and number of clusters as for COCA. We assigned weight zero to every missing observation (more details on how to use KLIC with incomplete data can be found in the Supplementary Material). The weighted consensus matrix is shown in Figure 5 (centre). The weights assigned on average to the observations in each dataset are as follows: copy number 31.4%, methylation 19.2%, miRNA 17.8%, mRNA 16.4% and protein 15.2%.

Similarly to what was observed by Hoadley *et al.* (2014), both the clusters obtained using COCA and KLIC correspond well with the tissue-of-origin classification of the tumours. However, there are a few differences between the two: the coincidence matrix is shown in Figure 5 (right). Further details on how we tried to replicate the data analysis of Hoadley *et al.* (2014) and how we applied KLIC to these data can be found in the Supplementary Material.

### 4.3 Transcriptional module discovery

**Fig. 6.**
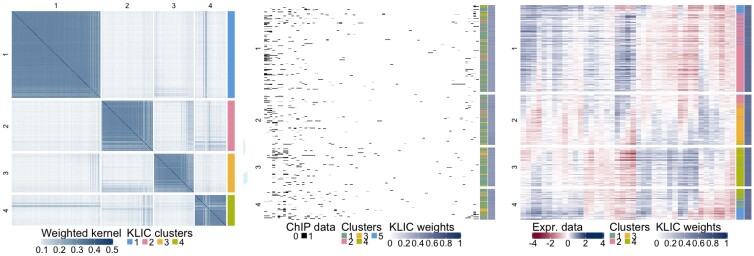
Transcriptional module discovery, KLIC output. Left: weighted kernel matrix obtained with KLIC, where each row and column corresponds to a gene, and final clusters. Centre: transcription factor data, where each row represents a gene and each column a transcription factor, black dots correspond to transcription factors that are believed to be able to bind to the promoter region of the corresponding gene with high confidence; clusters obtained using BHC on the transcription factor data and weight assigned by KLIC to each data point. Right: gene expression data, where each row is a gene and each column a time point, clusters obtained using PAM on the gene expression data, and weights assigned by KLIC to each data point

We clustered the 551 genes based on the gene expression and transcription factor data using KLIC. For each dataset, the consensus matrices were obtained as explained in Section 2.1. The clustering algorithms used in this step were partitioning around medoids (PAMs; Kaufman and Rousseeuw, 2009) with the correlations between data points as distances for the gene expression data and Bayesian hierarchical clustering (BHC) for the transcription factor data (Cooke *et al.*, 2011; Heller and Ghahramani, 2005). The consensus matrices obtained in this way were then used as input to KLIC. The algorithm was run with number of clusters ranging from 2 to 20. We found that the silhouette is maximized by setting the number of clusters to four. Figure 6 shows the weighted kernel matrix given by KLIC where the rows and columns are sorted by final cluster. Next to it are reported the data, where the observations are in the same order as in the kernel matrix. The clusters obtained independently on each dataset are also shown on the right of each plot. The kernel matrices of each dataset can be found in the Supplementary Material.

We also applied COCA to this dataset, with the initial clusters for each dataset obtained with the same clustering algorithms as those used for the consensus matrices. The metrics used to choose the number of clusters for the initial clustering of the expression data are reported in the Supplementary Material. BHC does not require the number of clusters to be set by the user. For the final clustering the number of clusters was chosen in order to maximize the silhouette, considering all values between 2 and 10. This resulted in choosing the 10-cluster solution.

In order to assess the quality of the clusters, we make use of the Gene Ontology Term Overlap (GOTO) scores of Mistry and Pavlidis (2008). Each score is an indication of the number of annotations that, on average, are shared by genes belonging to the same clusters. These are available for three different ontologies: biological process, molecular function and cellular component. More details on these scores and how they are calculated can be found in the Supplementary Material of Kirk *et al.* (2012). We report in Table 1, the GOTO scores of both KLIC and COCA clusters, for both number of clusters selected by KLIC (4) and COCA (10). We also show the scores obtained clustering each dataset separately. We observe that, while in the case of four clusters no information is lost by combining the datasets; by dividing data into 10 clusters one obtains more biologically meaningful clusters. Moreover, KLIC does a better job at combining the datasets, by better exploiting the information contained in the data and down-weighting the kernel of the ChIP dataset, which contains less information. More details about the kernel matrices and weights can be found in the Supplementary Material.

**Table:** Table 1. GOTO scores for different sets of data, clustering algorithms and numbers of clusters

Clusters	Dataset(s)	Algorithm	GOTO BP	GOTO MF	GOTO CC
8	ChIP	BHC	6.09	0.90	8.33
4	Expression	PAM	6.12	0.91	8.41
4	ChIP + Expr.	COCA	6.12	0.91	8.41
4	ChIP + Expr.	KLIC	6.12	0.91	8.41
10	ChIP + Expr.	COCA	6.28	0.93	8.51
10	ChIP + Expr.	KLIC	6.32	0.95	8.53

*Note*: ‘BP’ stands for Biological Process ontology; ‘MF’ for Molecular Function; and ‘CC’ for Cellular Component.

## 5 Discussion

In the first part of this work, we have given the algorithm for COCA, a widely used method in integrative clustering of genomic data, highlighting the main issues of using this method. We have also presented KLIC, a novel approach to integrative clustering, that allows multiple datasets to be combined to find a global clustering of the data and is well suited for the analysis of large datasets, such as those often encountered in genomics applications. A defining difference between KLIC and COCA is that, while COCA performs a combination of the clusters found in each dataset, KLIC uses the similarities between data points observed in each dataset to perform the integrative step. Moreover, KLIC weights each dataset individually, which allows more informative datasets to be up-weighted relative to less informative ones, as demonstrated in our simulation study. Finally, we have used KLIC to integrate multiple omic datasets, in two different real world applications, finding biologically meaningful clusters. The results compare favourably to those obtained with COCA.

## Funding

This work was supported by the UK Medical Research Council [MC_UU_00002/10 and MC_UU_00002/13] and the National Institute for Health Research [Cambridge Biomedical Research Centre at the Cambridge University Hospitals NHS Foundation Trust (The views expressed are those of the authors and not necessarily those of the NHS, the NIHR or the Department of Health and Social Care)]. Partly funded by the RESCUER project. RESCUER has received funding from the European Union's Horizon 2020 research and innovation programme under grant agreement No. 847912.

*Conflict of Interest*: none declared.

## Supplementary Material

btaa593_supplementary_data
