# Supplementary material for: Multiple kernel learning for integrative consensus clustering of omic datasets
Source: Bioinformatics. 2020 Jun 27;36(18):4789–96. doi: 10.1093/bioinformatics/btaa593 (PMC7750932; doi:10.1093/bioinformatics/btaa593)
Supplement: btaa593_supplementary_data [file btaa593_supplementary_data.pdf]

Multiple kernel learning for integrative consensus clustering of 'omic datasets

Alessandra Cabassi[1] and Paul D. W. Kirk[1,2]

[1] *MRC Biostatistics Unit*
[2] *Cambridge Institute of Therapeutic Immunology & Infectious Disease (CITIID)*
University of Cambridge, U.K.

Contents

S1 Methods **S2**
S1.1 Kernel k-means clustering S2
S1.2 How to use KLIC with incomplete data S3
S1.3 Algorithms S4

S2 Simulation study **S6**
S2.1 RBF kernel S6
S2.2 Additional simulation settings S8
S2.2.1 Datasets with nested clusters S8
S2.2.2 Comparison between KLIC, COCA, and other methods S10
S2.2.3 Sensitivity analysis S12

S3 Multiplatform analysis of 12 cancer types **S13**
S3.1 Replicating the analysis of Hoadley et al. (2014) S13
S3.2 Output of KLIC S18

S4 Transcriptional module discovery **S20**
S4.1 Clustering algorithms for the ChIP data S20
S4.1.1 Bayesian Hierarchical Clustering S20
S4.1.2 PAM with Gower's distance S20
S4.1.3 Greedy Bayesian non-parametric clustering algorithm S22
S4.2 Choice of the number of clusters S26

Bibliography **S28**

S1 Methods

S1.1 Kernel k-means clustering

Before moving on to the kernel k-means, we first describe the original k-means clustering algorithm (Steinhaus, 1956). Let $\boldsymbol{x}_1, \dots, \boldsymbol{x}_N$ indicate the observed dataset, with $\boldsymbol{x}_n \in \mathbb{R}^P$ and z_{nk} be the corresponding cluster labels, where $\sum_k z_{nk} = 1$ and $z_{nk} = 1$ if x_n belongs to cluster k, zero otherwise. We denote by Z the $N \times K$ matrix with ij-th element equal to z_{ij}. The goal of the k-means algorithm is to minimise the sum of all squared distances between the data points $\boldsymbol{x}_n$ and the corresponding cluster centroid $\boldsymbol{m}_k$. The optimisation problem is

$$\underset{Z}{\text{minimise}} \quad \sum_n \sum_k z_{nk} \|\boldsymbol{x}_n - \boldsymbol{m}_k\|_2^2 \tag{1a}$$

$$\text{subject to} \quad \sum_k z_{nk} = 1, \ \forall n, \tag{1b}$$

$$N_k = \sum_n z_{nk}, \ \forall k, \tag{1c}$$

$$\boldsymbol{m}_k = \frac{1}{N_k} \sum_n z_{nk} \boldsymbol{x}_n, \ \forall k. \tag{1d}$$

Now we can show how the kernel trick works in the case of the k-means clustering algorithm (Girolami, 2002). Redefining the objective function of Equation (1a) based on the distances between observations and cluster centres in the feature space $\mathcal{H}$, the optimisation problem becomes:

$$\underset{Z}{\text{minimise}} \quad \sum_n \sum_k z_{nk} \|\phi(\boldsymbol{x}_n) - \tilde{\boldsymbol{m}}_k\|_{\mathcal{H}}^2 \tag{2a}$$

$$\text{subject to} \quad \sum_k z_{nk} = 1, \ \forall n, \tag{2b}$$

$$N_k = \sum_n z_{nk}, \ \forall k, \tag{2c}$$

$$\tilde{\boldsymbol{m}}_k = \frac{1}{N_k} \sum_n z_{nk} \phi(\boldsymbol{x}_n), \ \forall k. \tag{2d}$$

where we indicated by $\tilde{\boldsymbol{m}}_k$ the cluster centroids in the feature space $\mathcal{H}$. Using this kernel, each term of the sum in Equation (2a) can be written as a function of $\delta(\boldsymbol{x}_i, \boldsymbol{x}_j)$. Therefore, there is no need to evaluate the map ϕ at every point $\boldsymbol{x}_i$ to compute the objective function of Equation (2a). Instead, one just needs to know the values of the kernel evaluated at each pair of data points $\delta(\boldsymbol{x}_i, \boldsymbol{x}_j)$, $i, j = 1, \dots, N$. This is what is commonly referred to as the kernel trick.

Defining L as the $K \times K$ diagonal matrix with kth diagonal element equal to N_k^{-1} and Δ the $N \times N$ matrix with ijth entry equal to $\delta(\boldsymbol{x}_i, \boldsymbol{x}_j)$, the optimisation problem (2) can be rewritten as a trace maximisation problem (Gönen and Margolin, 2014):

$$\underset{Z}{\text{maximise}} \quad \operatorname{tr}(L^{\frac{1}{2}} Z' \Delta Z L^{\frac{1}{2}}) \tag{3a}$$

$$\text{subject to} \quad Z 1_k = 1_n, \tag{3b}$$

$$z_{nk} \in \{0, 1\}, \quad \forall n, k. \tag{3c}$$

The integrality constraints make this problem difficult to solve. However, the corresponding linear problem obtained by relaxing the integer constraints of Equation (3c) to $0 \leq z_{nk} \leq 1$ for all n, k can be solved by performing kernel PCA on the kernel matrix Δ and setting the matrix $H = Z L^{\frac{1}{2}}$ to

the K eigenvectors that correspond to K largest eigenvalues (Schölkopf et al., 1998). The clustering solution can be found by first normalising all rows of H to be on the unit sphere and then performing k-means clustering on the normalised matrix. Other possible approaches to derive a final clustering from H are listed in Shawe-Taylor and Cristianini (2004).

S1.2 How to use KLIC with incomplete data

This section is dedicated to giving further details about how missing data can be handled by using KLIC. The strategy explained in this section was used in the application of KLIC to the multiplatform analysis of 12 cancer types in Section 4.2 of the main paper.

The optimisation problem that is solved to find the optimal clustering and weights in localised multiple kernel k-means is:

$$\begin{aligned} \underset{H,\Theta}{\text{maximise}} \quad & \text{tr}(H'\Delta_\Theta H) - \text{tr}(\Delta_\Theta) && \text{(4a)} \\ \text{subject to} \quad & H'H = 1_k, && \text{(4b)} \\ & \Theta' 1_M = 1, && \text{(4c)} \\ & \Delta_\Theta = \sum_m (\boldsymbol{\theta}_m \boldsymbol{\theta}'_m) \circ \Delta_m, && \text{(4d)} \end{aligned}$$

where $\circ$ is the Hadamard product. As stated in Section 2.2.2 of the main paper, one can optimise the objective function of Equation (4a) with a two-step procedure, that iteratively (1) solves a standard kernel k-means problem with kernel δ_Θ, keeping the weight matrix Θ fixed and then (2) optimises the objective function with respect to Θ. Again, the first step reduces to solving one optimisation problem with a single kernel (Equations 3) and in the second step one just needs to solve a quadratic programming (QP) problem. In particular, the QP problem in step (2) is:

$$\begin{aligned} \underset{\Theta}{\text{minimise}} \quad & \sum_{m=1}^{M} \boldsymbol{\theta}_m^T ((I_n - HH^T) \circ \Delta_m) \boldsymbol{\theta}_m && \text{(5a)} \\ \text{subject to} \quad & \Theta \in \mathbb{R}_+^{N \times M}, && \text{(5b)} \\ & \Theta' 1_M = 1_N. && \text{(5c)} \end{aligned}$$

Now, if some of the observations are missing in some of the datasets, we can define by $I_m \subset \{1, \dots, N\}$ the set of the missing values in each dataset $m = 1, \dots, M$ and make sure that the corresponding kernel Δ_m is such that

$$\begin{aligned} \Delta_{ij}^m &= 0 \quad \forall i \in I_m, j \neq i, \\ \Delta_{ii}^m &= 1 \quad \forall i \in I_m. \end{aligned}$$

The resulting matrix Δ_m is a weighted sum of co-clustering matrices with structure

$$\Delta_m = \begin{bmatrix} \Delta'_m & 0 & 0 & 0 \\ 0 & 1 & 0 & 0 \\ 0 & 0 & \ddots & 0 \\ 0 & 0 & 0 & 1 \end{bmatrix},$$

where Δ'_m is the m-th kernel matrix for the available data and the observations are ordered such that the missing ones are at the bottom of the matrix for presentational purposes. Therefore, it is a valid kernel matrix.

Moreover, it is possible to cancel the influence the missing observations on the final solutions by setting their weight to zero in optimisation problem (5):

$$
\begin{aligned}
\underset{\Theta}{\text{minimise}} \quad & \sum_{m=1}^{M} \boldsymbol{\theta}_m^T((I_n - HH^T) \circ \Delta_m)\boldsymbol{\theta}_m && \text{(6a)} \\
\text{subject to} \quad & \Theta \in \mathbb{R}_+^{N \times M}, && \text{(6b)} \\
& \Theta' 1_M = 1_N, && \text{(6c)} \\
& \theta_{mi} = 0 \qquad \forall i \in I_m,\ m = 1, \dots, M. && \text{(6d)}
\end{aligned}
$$

This corresponds to adding $|I_1| + \cdots + |I_M|$ equality constraints, each one on a different variable, or, equivalently, to removing a number $|I_1| + \cdots + |I_M|$ of variables from the optimisation problem. Therefore, (6) is a QP problem. The objective function (4) can then be minimised by iterating between steps (1) and (2) as in the previous case, with the additional constraints (6d) in step (2).

S1.3 Algorithms

Algorithm 1: Consensus cluster (CC).

Input : Dataset X, number of clusters K.
Initialise : Consensus matrix $\Delta^K = 0_{N \times N}$.
Matrix of resampling counts $D_{ij} = 0_{N \times N}$.

for $h \in \{1, \dots, H\}$ **do**
  $X^{(h)}$ = resample from the rows and/or columns of X
  $\mathbf{c}^{(h)}$ = divide the items of $X^{(h)}$ into K clusters
  $C^{(h)}$ = build the co-clustering matrix corresponding to $\mathbf{c}^{(h)}$
  for $i, j \in \{1, \dots, n\}$ **do**
    $\Delta_{ij}^K = \Delta_{ij}^K + C_{ij}^{(h)}$
    $D_{ij} = D_{ij} + \mathbb{1}_{ij}^{(h)}$
  end
end
for $i, j \in \{1, \dots, n\}$ **do**
  $\Delta_{ij}^K = \Delta_{ij}^K / \min\{D_{ij}, 1\}$
end
Output : Consensus matrix Δ^K.

Algorithm 2: Cluster of clusters analysis (COCA)

Input : M datasets X_m
Number of clusters K_m in each dataset
Global number of clusters K.
Initialise : MOC $= 0_{\bar{K}\times N}$.
for $m \in \{1, \ldots, M\}$ **do**
  $\mathbf{c}^m$ = cluster the items in dataset X_m into K_m clusters
  for $n \in \{1, \ldots, N\}$, $k \in \{1, \ldots, K_m\}$ **do**
    Set $MOC_{n,m_k} = 1$ if $\mathbf{c}_i^m = k$
  end
end
for $h \in \{1, \ldots, H\}$ **do**
  $MOC^{(h)}$ = resample from the rows and/or columns of MOC
  $\mathbf{c}^{(h)}$ = divide the items of $X^{(h)}$ into K clusters
  $C^{(h)}$ = build the co-clustering matrix corresponding to $\mathbf{c}^{(h)}$
  for $i, j \in \{1, \ldots, n\}$ **do**
    $\Delta_{ij} = \Delta_{ij} + C_{ij}^{(h)}$
    $D_{ij} = D_{ij} + \mathbb{I}_{ij}^{(h)}$
  end
end
for $i, j \in \{1, \ldots, n\}$ **do**
  $\Delta_{ij} = \Delta_{ij} / \min\{D_{ij}, 1\}$
end
Find final clustering $\boldsymbol{c}^K$ using hierarchical clustering on Δ^K.
Output : Cluster labels $\boldsymbol{c}^K$.

Algorithm 3: KLIC: Kernel Learning Integrative Clustering

Input : M datasets X_m
Maximum number of clusters K.
for $m \in \{1, \ldots, M\}$ **do**
  Δ_m = compute kernel for X_m
end
for $k \in \{1, \ldots, K\}$ **do**
  $[\boldsymbol{w}_k, \boldsymbol{c}_k]$ = apply multiple kernel k-means to $\Delta_1, \ldots, \Delta_M$
  $\boldsymbol{s}_k$ = calculate average silhouette of $\boldsymbol{c}_k$
end
Choose k such that $s_k \geq s_j, \forall j \neq k$.
return k, $\boldsymbol{w}_k$, $\boldsymbol{c}_k$.
Output : Best number of clusters k
Set of kernel weights $\boldsymbol{w} = [w_1, \ldots, w_M]$
Cluster labels $\boldsymbol{c} = [c_1, \ldots, c_N]$

S2 Simulation study

S2.1 RBF kernel

The RBF kernel is defined as

$$\delta(\mathbf{x}, \mathbf{x}') = \exp\left\{-\frac{\|\mathbf{x} - \mathbf{x}'\|^2}{2\sigma^2}\right\}, \quad (7)$$

where $\boldsymbol{x}, \boldsymbol{x}' \in \mathbb{R}^P$, $\|\cdot\|$ is the Euclidean distance and the parameter σ is the so-called *characteristic length scale*. In order to find the best possible value of σ for each synthetic dataset, we generate 100 dataset for each value s (the parameter that indicates the separation between cluster means) considered in our simulation setting, which are as follows:

- $s = 1.5$ in setting 1 (similar datasets);
- $s = 0, 1, 2, 3$ in setting 2 (datasets with different levels of noise);
- $s = 0, 0.5, 1, 1.5, 2, 2.5, 3, 3.5, 4$ in the additional simulation settings presented below (Section S2.2).

For each dataset, we build one kernel for each of the following values of σ: 0.001, 0.005, 0.01, 0.05, 0.1, 0.5, 1, 5, 10, 50. We then use kernel k-means to cluster the data and compute the ARI between the clustering obtained in this way and the true cluster labels (Figure S2). We then choose the value of σ maximising the average ARI for each value of s.

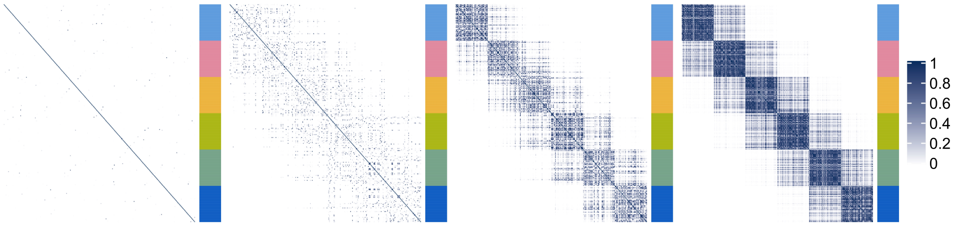

Figure S1. Kernels obtained for the same datasets as those used for Figure 1 (first row) in the main paper, using RBF kernels.

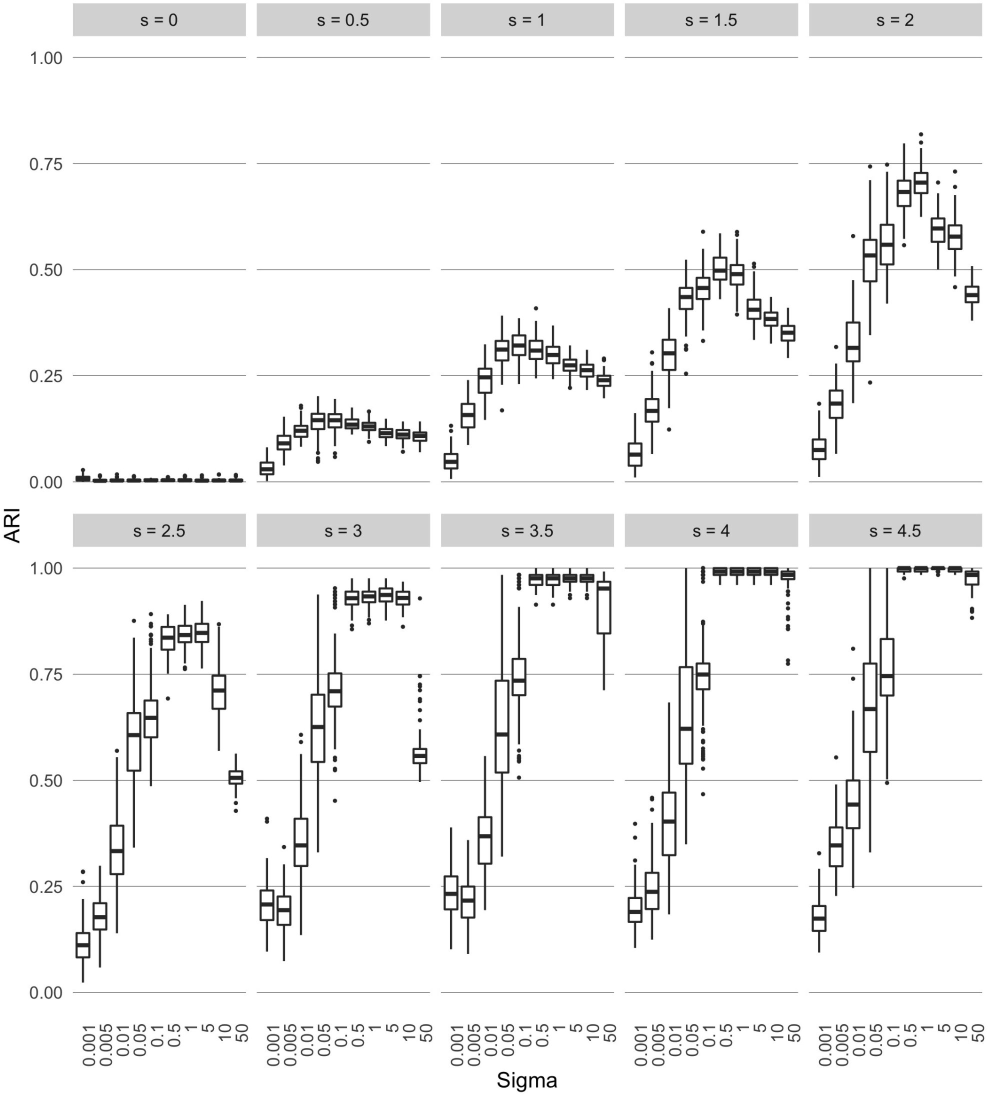

Figure S2. ARI between the clusters obtained with an kernel k-means on RBF kernels for different values of the characteristic length scale parameter and separation between clusters.

S2.2 Additional simulation settings

We present here some additional simulation settings that were omitted from the main paper for the sake of brevity.

S2.2.1 Datasets with nested clusters

We investigate how the algorithm copes with the ambiguous situation of nested clusters. To this end, we generate two datasets with the same value of the parameter s setting the distance between cluster centres. The first one has six clusters, while the second one only has three clusters, each of them containing two of the clusters of the other dataset (Figure S3).

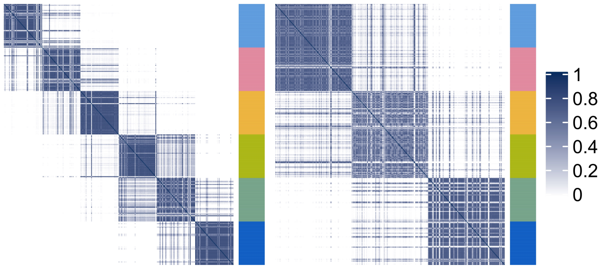

Figure S3. Consensus matrices of the synthetic data. Blue indicates high similarity. The colours of the bar to the right of each matrix indicate the cluster labels. Consensus matrices of two datasets with nested clusters: the one on the left has six clusters, whereas the one on the right has three clusters formed by merging two of the clusters of the dataset with six clusters.

Since the algorithm works only with a fixed number of clusters, we try both with $K = 3$ and $K = 6$. The ARI and the average weights assigned to each matrix are reported in Figure S5. For $K = 6$, the weights assigned to each matrix are not as we expected: the matrix with three clusters is weighted slightly more highly than the other one. To investigate this phenomenon, we introduce an additional way to score how strong the signal is in each dataset. We use the *cophenetic correlation coefficient*, a measure of how faithfully hierarchical clustering would preserve the pairwise distances between the original data points (Brunet et al., 2004, Sokal and Rohlf, 1962). Given a dataset $X = [\boldsymbol{x}_1, \boldsymbol{x}_2, \dots, \boldsymbol{x}_N]$ and a similarity matrix $\Delta \in \mathbb{R}^{N \times N}$, we define the *dendrogrammatic distance* between $\boldsymbol{x}_i$ and $\boldsymbol{x}_j$ as the height of dendrogram at which these two points are first joined together by hierarchical clustering and we denote it by η_{ij}. The cophenetic correlation coefficient ρ is calculated as

$$\rho = \frac{\Sigma_{i<j}(\Delta_{ij} - \bar{\Delta})(\eta_{ij} - \bar{\eta})}{\sqrt{\Sigma_{i<j}(\Delta_{ij} - \bar{\Delta})\Sigma_{i<j}(\eta_{ij} - \bar{\eta})}}, \tag{8}$$

where $\bar{\Delta}$ and $\bar{\eta}$ are the average values of Δ_{ij} and η_{ij} respectively. The cophenetic correlation coefficient of a consensus matrix can be interpreted as an indication of the level of its dispersion or, equivalently, of the stability of the clustering used in CC. If the clusters are invariant under subsampling of the data features/observations, then the consensus matrix has all entries equal to either one or zero, and cophenetic correlation coefficient equal to one. On the other hand, if clusters vary at each iteration of consensus clustering, the entries of the consensus matrix are scattered between zero and one, and the corresponding cophenetic correlation coefficient is negative. The consensus matrices shown in Figure 1 of the main paper, for instance, have increasing cophenetic

correlation going from left (lower cluster separability) to right (higher cluster separability). We find that in this case the consensus matrices with $K = 3$ have slightly higher cophenetic correlation than the ones with $K = 6$ with the same level of cluster separability s. This explains why higher weights are assigned to the former. This suggests that, in ambiguous cases, localised kernel k-means assigns higher weights (on average) to the kernels with highest cophenetic correlation. Intuitively, the sum of within-cluster distances in the feature space is zero when each pair of data points has similarity one if both data points are in the same cluster, and zero otherwise. Minimising that sum thus corresponds to finding the weights and cluster allocations that lead to a weighted kernel that is as close as possible to a kernel with cophenetic correlation one.

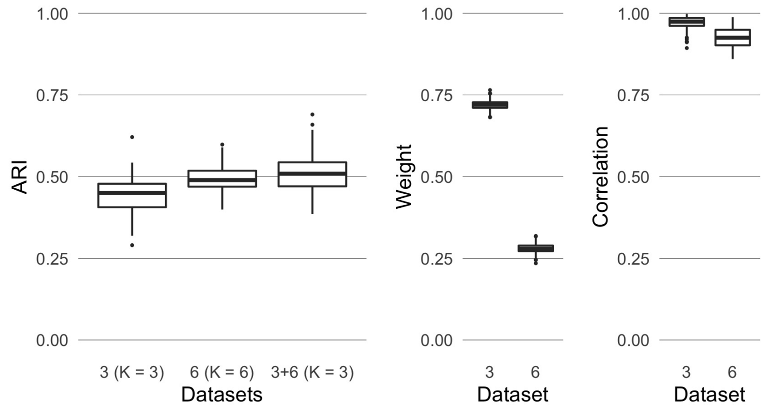

(a) True number of clusters for CC, $K = 3$ for global clustering.

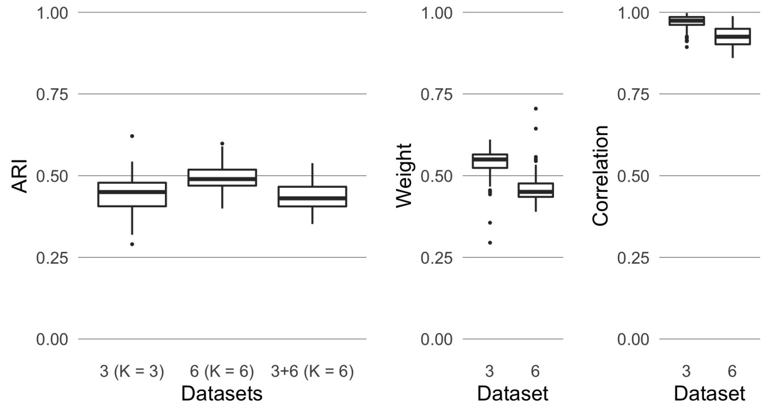

(b) True number of clusters for CC, $K = 6$ for global clustering.

Figure S4. Results of applying KLIC to datasets that have nested clusters. Left: ARI of KLIC applied to the datasets with three and six clusters separately (columns "3" and "6" respectively) and to those two datasets combined (column "3+6"). Centre: the weights assigned to each dataset. Right: cophenetic correlation coefficients of the consensus matrices built with $K = 3$ (for the dataset with three clusters) and $K = 6$ (for the dataset with six clusters). Higher weights are given to the kernels with higher cophenetic correlation, irrespectively of their number of clusters.

We also report the results obtained setting either $K = 3$ or $K = 6$ at each step of KLIC, i.e. consensus clustering of each dataset and MKL.

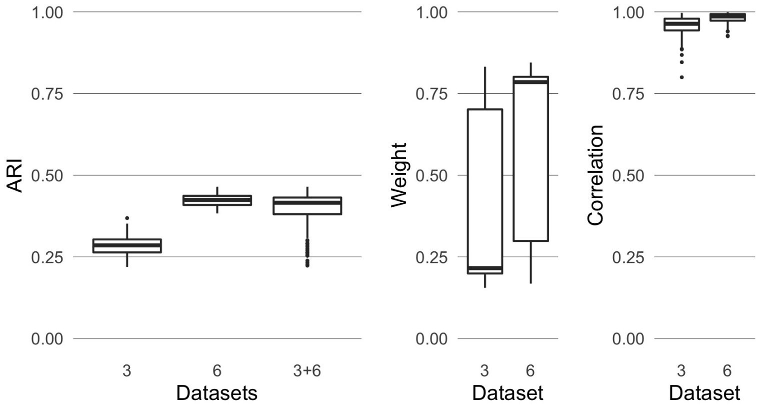

(a) $K = 3$ at each step.

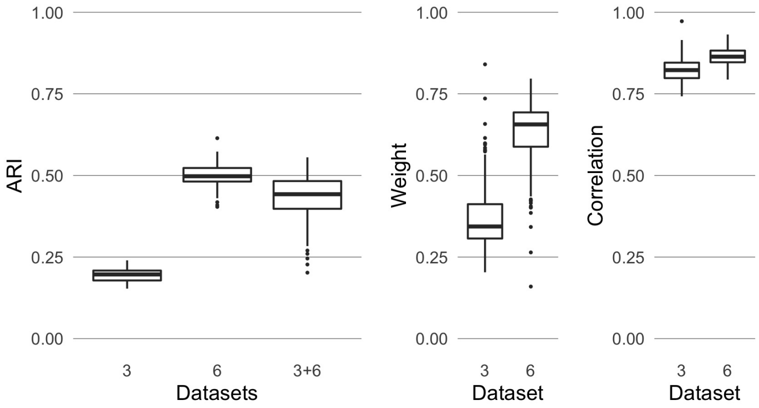

(b) $K = 6$ at each step.

Figure S5. Results of applying KLIC to datasets that have nested clusters. Left: ARI of KLIC applied to the datasets with three and six clusters separately (columns "3" and "6" respectively) and to those two datasets combined (column "3+6"). Centre: the weights assigned to each dataset. Right: cophenetic correlation coefficients of the consensus matrices built with $K = 3$ (top) and $K = 6$ (bottom). Higher weights are given to the kernels with higher cophenetic correlation, irrespectively of their number of clusters.

S2.2.2 Comparison between KLIC, COCA, and other methods

For simulation setting 1 (four datasets with the same level of cluster separability) only the results obtained with $s = 1.5$ are reported in the main paper. For completeness, we show here the corresponding figures for a range of other values of s in Figure S6.

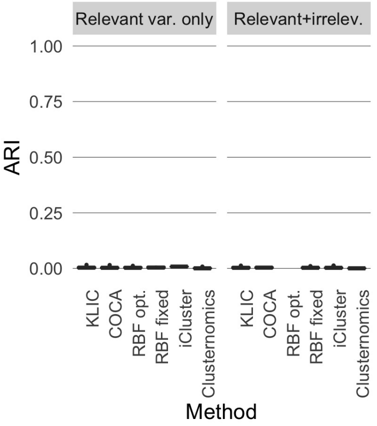

(a) $s = 0$.

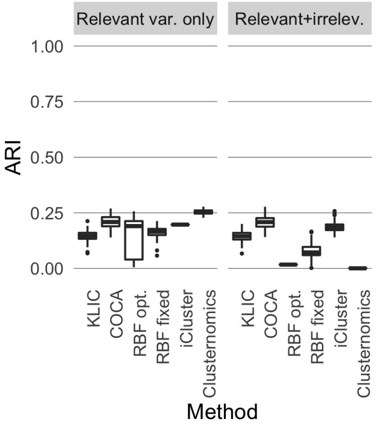

(b) $s = 0.5$.

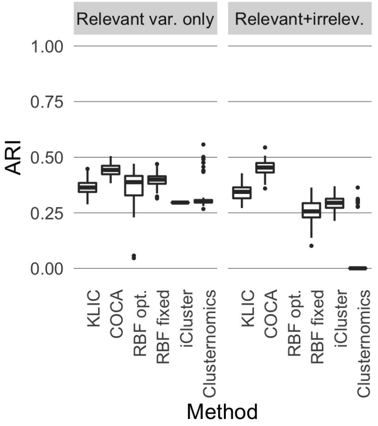

(c) $s = 1$.

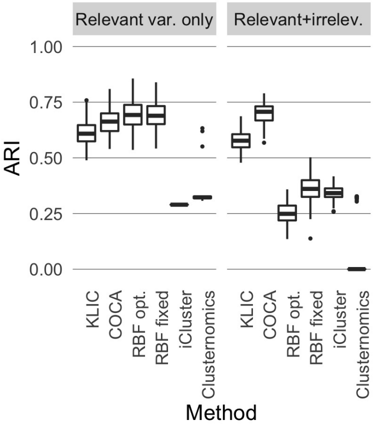

(d) $s = 1.5$.

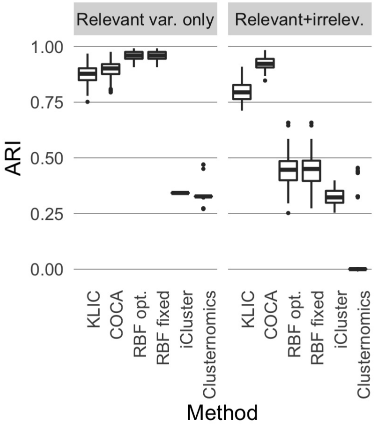

(e) $s = 2$.

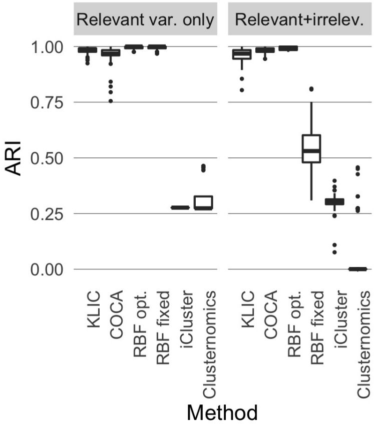

(f) $s = 2.5$.

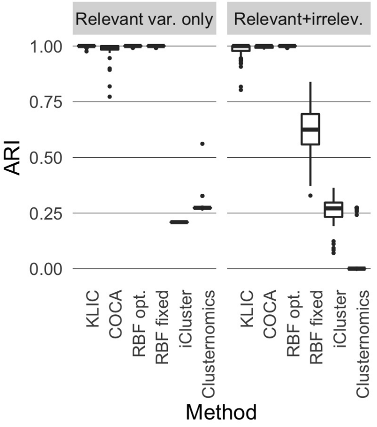

(g) $s = 3$.

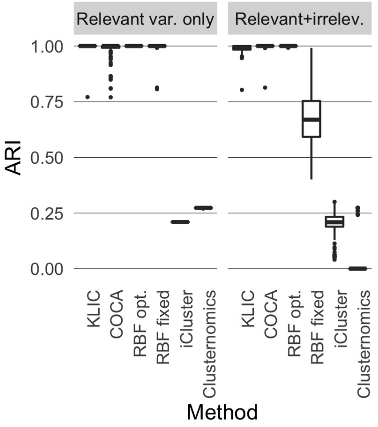

(h) $s = 3.5$.

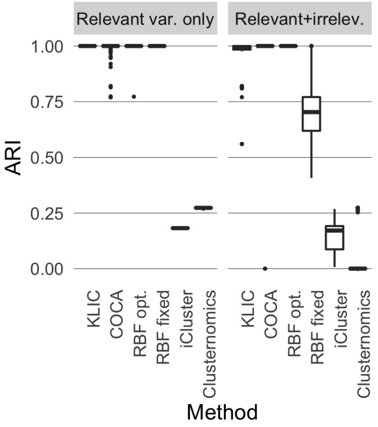

(i) $s = 4$.

Figure S6. Comparison between KLIC, COCA, and other clustering algorithms. ARI obtained using four datasets having the same clustering structure and cluster separability (as in Figure 2).

S2.2.3 Sensitivity analysis

The results presented in the main paper were obtained with the parameter `nstart` of the `kmeans` function (which determines the number of random initialisations of the algorithm) set to one both for KLIC and COCA. Figure S7 shows the ARI obtained for the same simulation settings as in Figure 4 in the main paper, with the `nstart` parameter set to 20. The figure shows that COCA is quite sensitive to the choice of this parameter, while KLIC is not. This explains the difference observed in Figure 4 of the main paper between the ARI of COCA obtained when using k-means and sparse k-means, since those two methods have different default values of `nstart`.

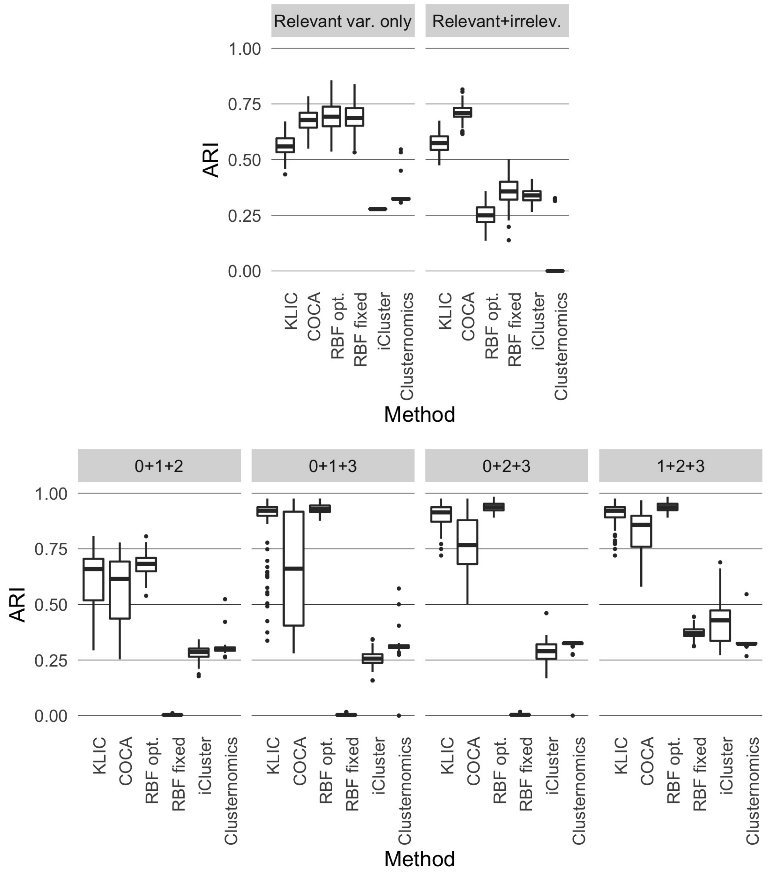

Figure S7. Comparison between KLIC, COCA, and other clustering algorithms. The labels ‘RBF opt.’ and ‘RBF fixed’ refer to the MKL method using an RBF kernel with either σ optimised or fixed at 1. Top: ARI obtained with each clustering algorithm using four datasets having the same clustering structure and cluster separability (as in Figure 2 in the main paper). Bottom: ARI obtained with COCA and KLIC for each of the subsets of heterogeneous datasets considered in Figure 3 in the main paper. The high ARI obtained with KLIC in all settings shows the advantage of using this method, especially when some of the datasets are noisy.

S3 Multiplatform analysis of 12 cancer types

In Section S3.1 we explain the steps we took to try to replicate the data preprocessing and cluster analysis of Hoadley et al. (2014). In Section S3.2 we give more details on the input and output of KLIC for this particular application.

S3.1 Replicating the analysis of Hoadley et al. (2014)

For each type of data we followed as closely as possible the procedures presented in the supplementary material of Hoadley et al. (2014). We present here the steps that we followed. The malignancies and corresponding acronyms considered in this study are: glioblastoma multiforme (GBM), serous ovarian carcinoma (OV), colon (COAD) and rectal (READ) adenocarcinomas, lung squamous cell carcinoma (LUSC), breast cancer (BRCA), acute myelogenous leukemia (AML), endometrial cancer (UCEC), renal cell carcinoma (KIRC), and bladder urothelial adenocarcinoma (BLCA). The agreement between the clustering analysis presented here and the clustering presented in the original Hoadley *et al.* paper ranged from excellent (for the protein and mRNA datasets) to quite poor (for the miRNA dataset).

Protein expression We used hierarchical clustering with Ward's agglomeration method and Pearson's correlation as the distance. Our clusters match exactly those of Hoadley *et al.* (i.e. the ARI is equal to one, see Figure S8).

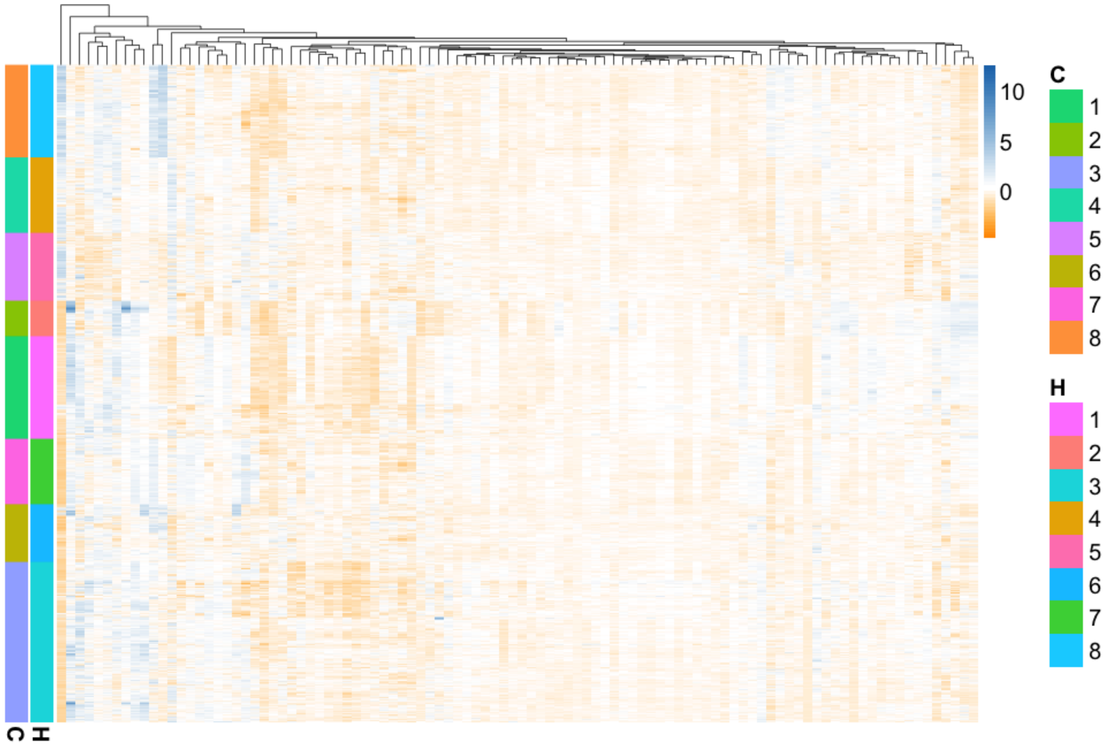

Figure S8. Protein expression clusters. High values are indicated in blue and low values in orange. C: Clusters found in this analysis. H: Clusters found in the original analysis of Hoadley *et al.* Adjusted Rand index between C and H: 1.

mRNA expression For mRNA expression, we proceeded as indicated by Hoadley et al. (2014). We chose the genes present in 70% of samples and then selected the 6,000 most variable genes. Then we used the ConsensusClusterPlus R package with settings `maxK=20`, `innerLinkage ="average"` `finalLinkage="average"`, `distance="pearson"`, `corUse="pairwise.complete.obs"`. The ARI is 0.917 (see Figure S9).

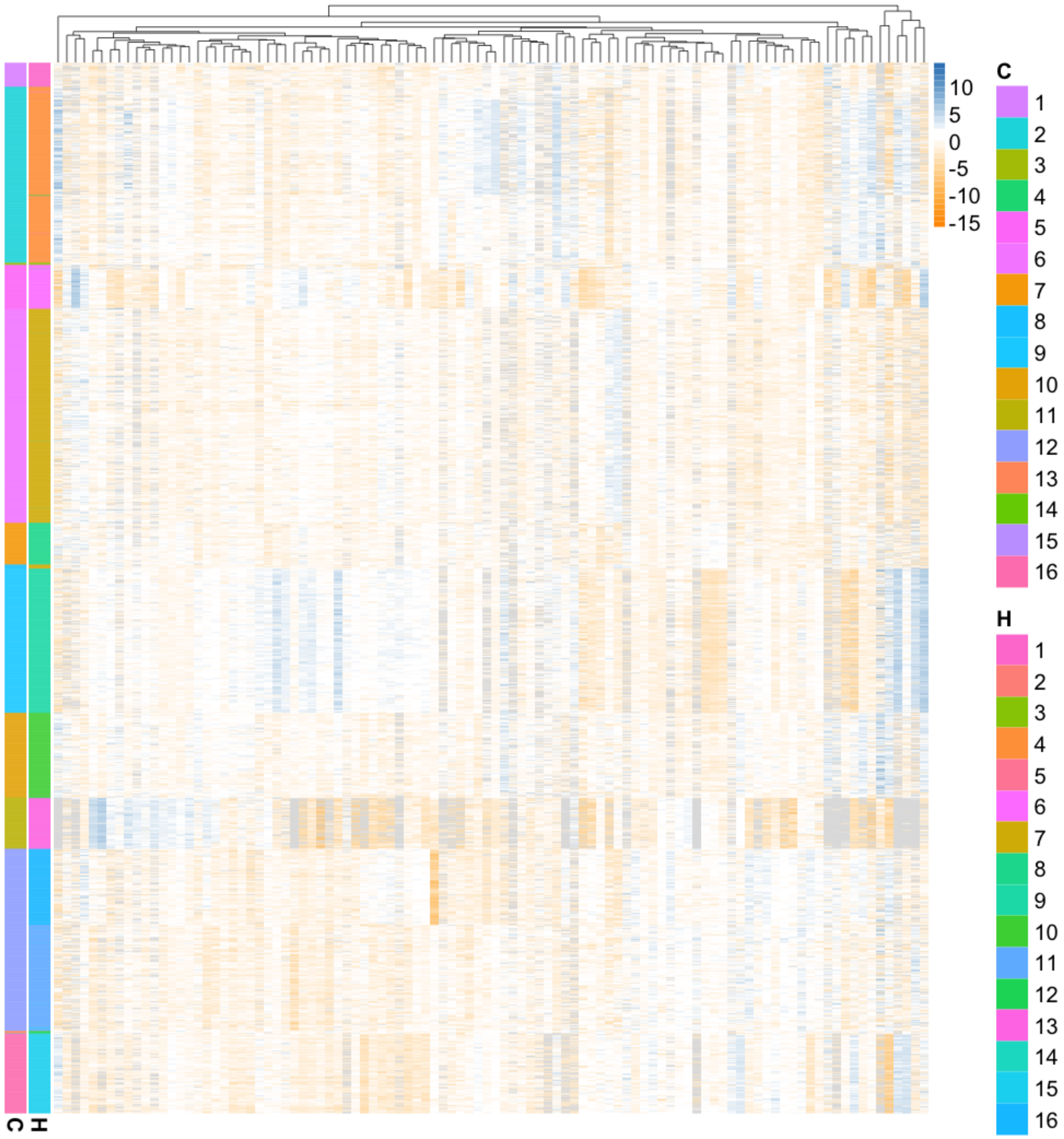

Figure S9. mRNA expression clusters. High values are indicated in blue and low values in orange. The dataset contains 600 genes but here we show only 100 of them. C: Clusters found in this analysis. H: Clusters found in the original analysis of Hoadley *et al.* Adjusted Rand index between C and H: 0.917.

DNA methylation We used hierarchical clustering with Jaccard's distance and Ward's agglomeration method. Hoadley et al. (2014) chose to divide the data into 19 clusters, so we did the same. Comparing our clusters to those of Hoadley et al. (2014), we obtained an ARI of 0.680 (see Figure S10).

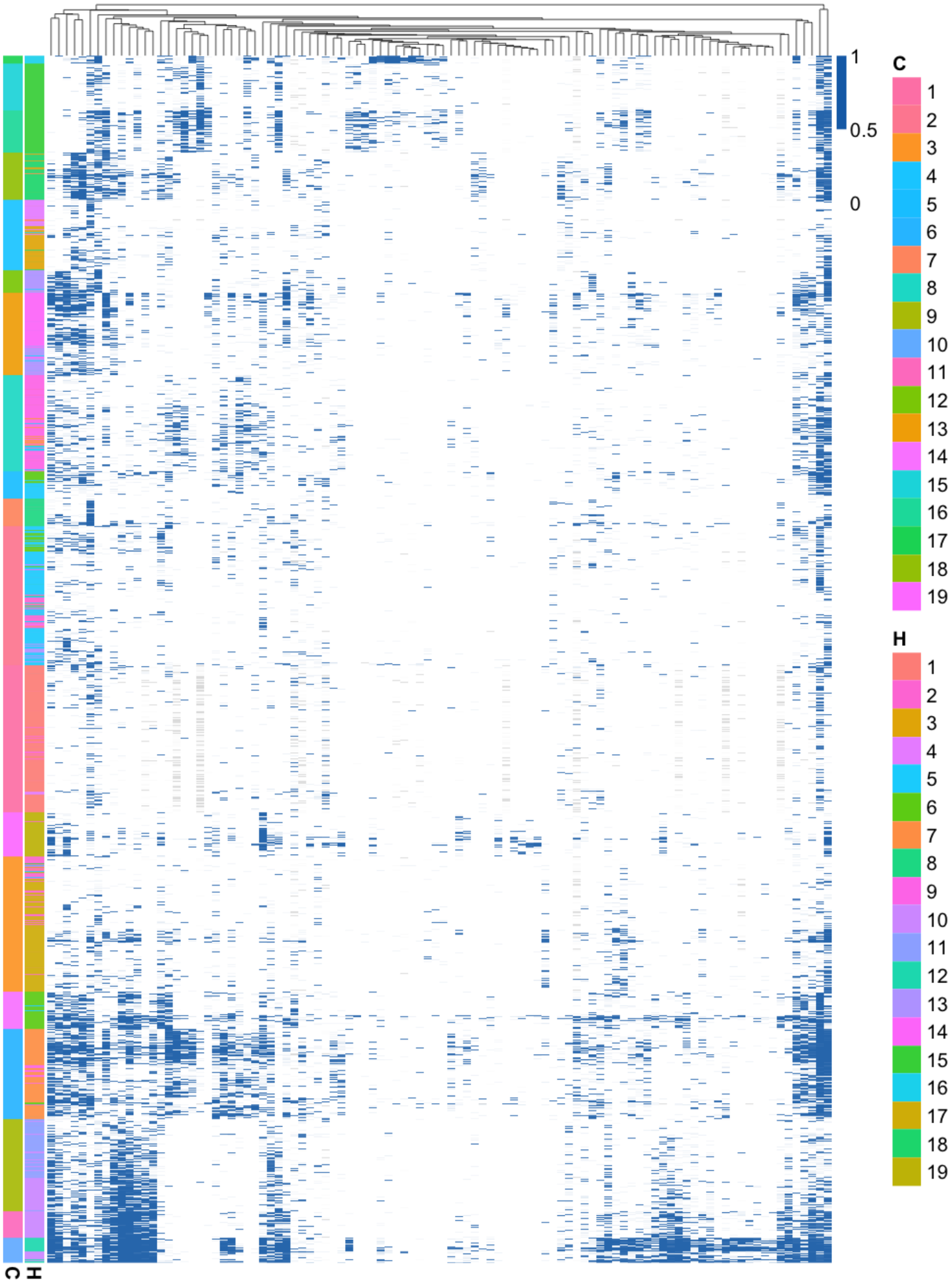

Figure S10. DNA methylation clusters. Blue cells correspond to methylated loci. Missing values are indicated in grey colour. Only 100 CpG loci are shown here, but the full dataset contains 2,043. C: Clusters found in this analysis. H: Clusters found in the original analysis of Hoadley *et al.* Adjusted Rand index between C and H: 0.680.

DNA copy number The clusters for the somatic copy number dataset were found using hierarchical clustering with Euclidean distance and Ward's method. The number of clusters was set to eight in the original manuscript based on the cophenetic distances and therefore we did the same here. The adjusted Rand index (ARI) comparing the clustering found in the present analysis with the clustering found in the original analysis of Hoadley *et al.* is 0.333 (see Figure S11).

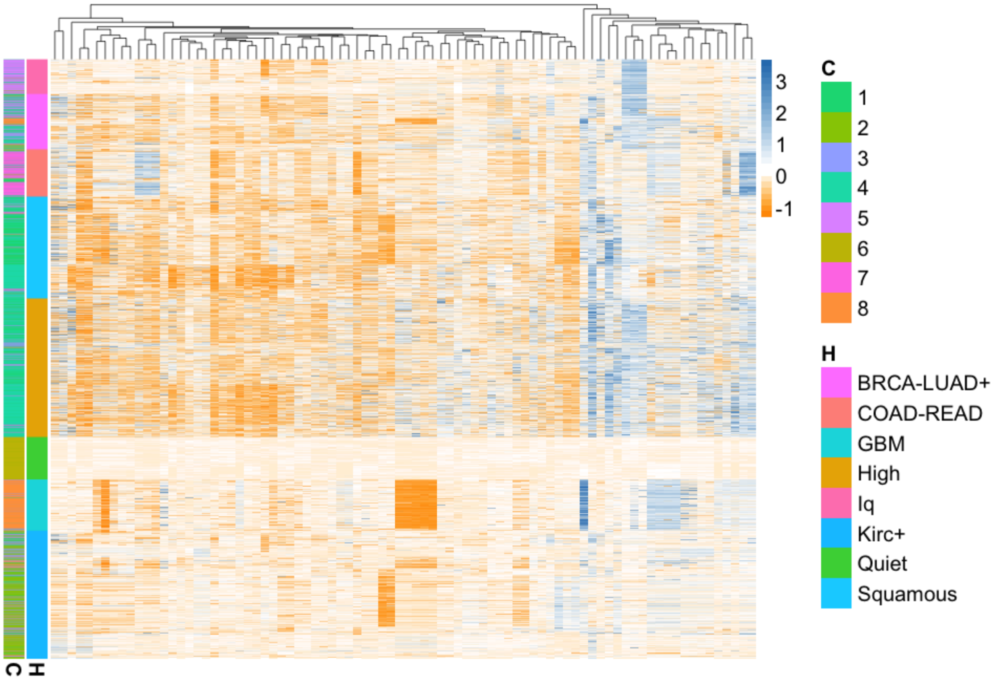

Figure S11. Somatic copy number clusters. High values are indicated in blue and low values in orange. C: Clusters found in this analysis. H: Clusters found in the original analysis of Hoadley *et al.* Adjusted Rand index between C and H: 0.333.

microRNA expression In the original manuscript the clusters of the microRNA-seq data were determined using a software program called *Cluster 3* (De Hoon et al., 2004). The same software was used to scale the data. Since it is was not possible to retrieve the clusters presented in the paper using this software, we used R to scale the data as was done by Cluster 3, namely applying a logarithmic transformation to the data and then median-centring. We found the final clusters using agglomerative hierarchical clustering in R (`agnes` command. We selected the number of clusters that maximises the silhouette, which is eight. The ARI is 0.255 (see Figure S12).

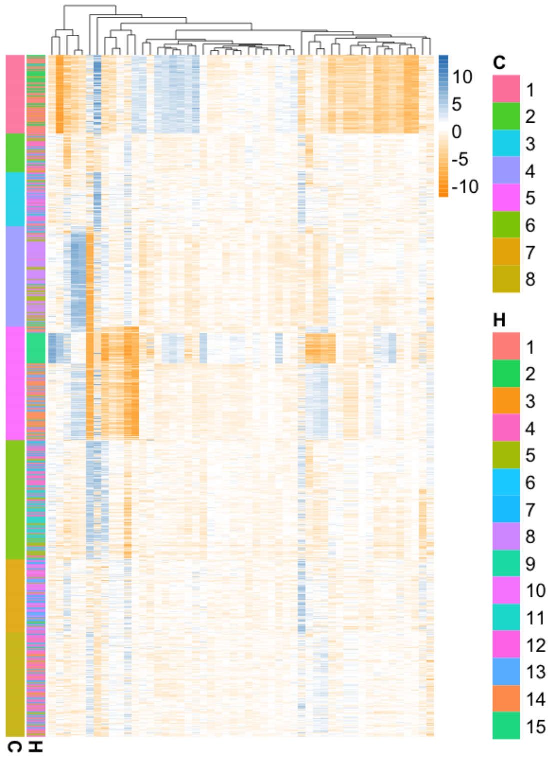

(a) Clusters. High values are indicated in blue, low values in orange.

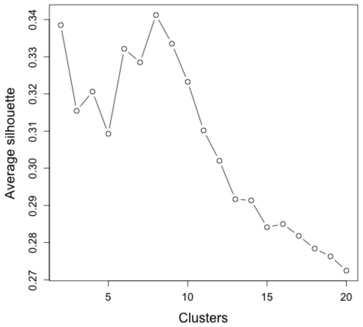

(b) Silhouette.

Figure S12. microRNA expression. C: Clusters found in this analysis. H: Clusters found in the original analysis of Hoadley *et al.* Adjusted Rand index between C and H: 0.255.

S3.2 Output of KLIC

The kernels corresponding to each dataset are shown in Figure S13, for each of them we also report the cophenetic correlation coefficient. Figure S14a shows the weights associated to each observation in each dataset. Figure S14b shows the average silhouette for all the number of clusters considered: the optimal values are between six and ten. Finally, Figure S14c shows the correspondences between the clusters obtained using KLIC and the tumour tissues. Most clusters correspond quite well with one or two tissue types (e.g. cluster 10 contains almost exclusively samples of renal cell carcinoma and cluster 6 contains colon and rectal adenocarcinomas), but not all.

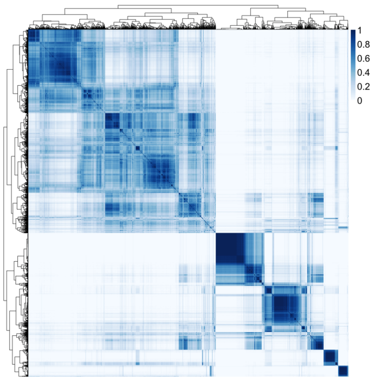

(a) DNA copy number. Cophenetic correlation coefficient: 0.736.

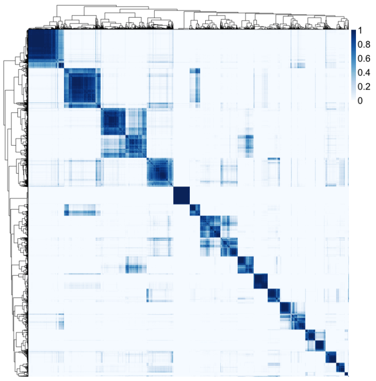

(b) DNA methylation. Cophenetic correlation coefficient: 0.859.

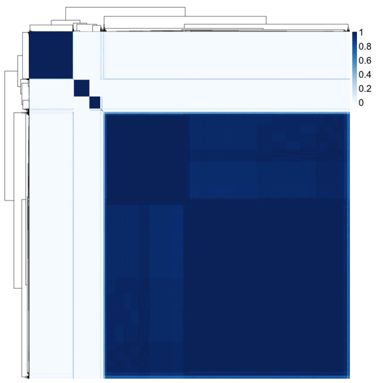

(c) mRNA expression. Cophenetic correlation coefficient: 0.974.

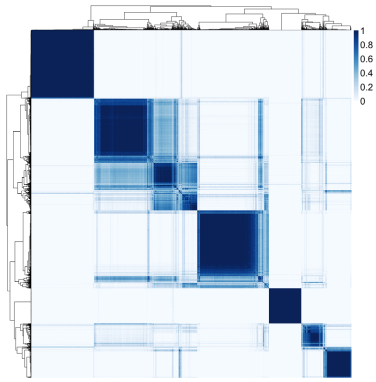

(d) miRNA expression. Cophenetic correlation coefficient: 0.923.

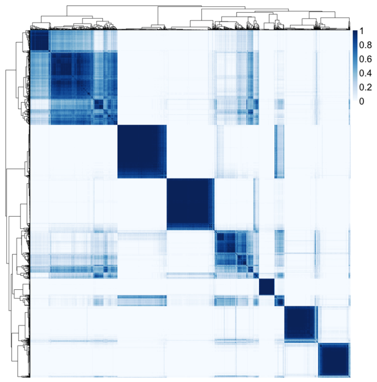

(e) Protein expression. Cophenetic correlation coefficient: 0.888.

Figure S13. Kernel matrices.

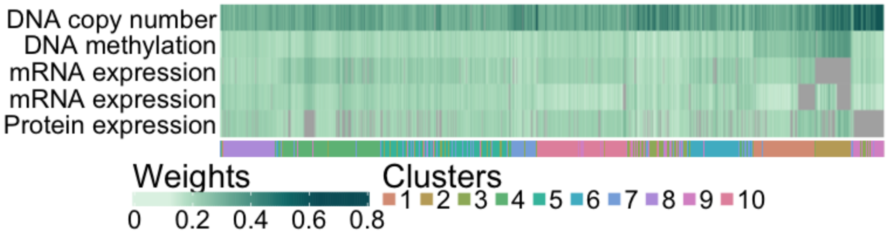

(a) Weights.

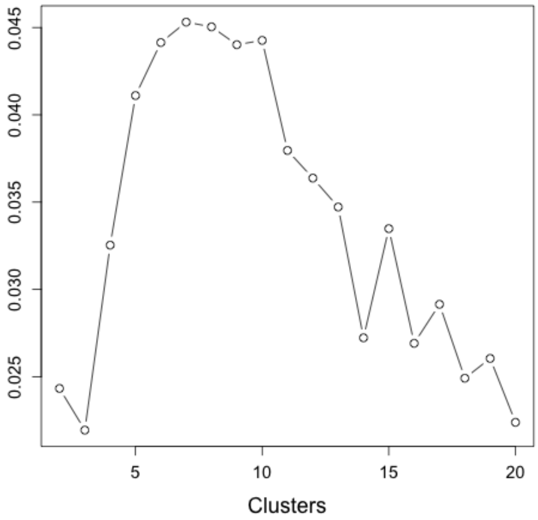

(b) Average silhouette.

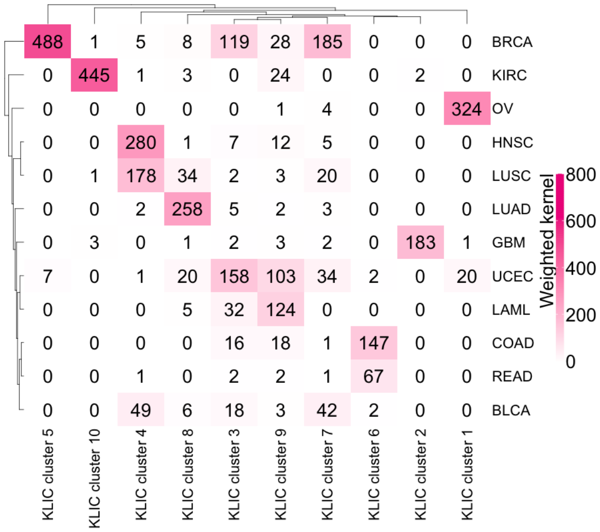

(c) Matrix of coincidences.

Figure S14. Output of KLIC. (a) Weights. Low weights are indicated in white and higher weights in green. Grey cells correspond to missing values, which have zero weight. (b) Average silhouette. The maximum is obtained for seven clusters. All numbers of clusters comprised between six and ten have similar values. (c) Matrix showing the correspondences between the clusters obtained by using KLIC and the tumour tissues.

S4 Transcriptional module discovery

This section is structured as follows. First, we give further details regarding the application of KLIC and COCA to transcriptional module discovery using Bayesian Hierarchical Clustering as the clustering algorithm for the ChIP data. Then, we consider other algorithms that could have been applied to this dataset and compare the new results with those reported in the main paper. Finally, we give more details about the choice of the number of clusters for PAM.

S4.1 Clustering algorithms for the ChIP data

The ChIP dataset is quite sparse. The data were discretised so that only transcription factors that are believed with high confidence to be able to bind to a gene's promoter region are marked as "ones"; all the others are "zeros". For this reason, in addition to BHC, we considered two clustering algorithms that are able to take into account this feature of the data. However, we show in Sections S4.1.2 and S4.1.3 that these methods often cluster genes with few transcription factors (i.e. observations for which most variables are zero) together, while the other genes end up in separate small clusters that are less stable under subsampling of the data. This leads to consensus matrices that have high cophenetic correlation coefficients but carry little information. We show that combining the corresponding kernels to that of the expression data does not always give more meaningful clustering solutions than those obtained on each data type separately. This highlights the importance of the kernel matrices as an intermediate diagnostic tool for KLIC, which can help choosing the right clustering algorithms.

S4.1.1 Bayesian Hierarchical Clustering

Bayesian Hierarchical Clustering (BHC; Heller and Ghahramani, 2005) is a method for agglomerative hierarchical clustering. The idea is that, similarly to classical agglomerative clustering algorithms, at the start each data point is considered as a different cluster; then, at each step, two clusters are merged. The main difference between classical hierarchical clustering and BHC is that in BHC merging is done based on Bayesian hypothesis testing, where the alternative hypotheses are "all data in clusters c_i and c_j were generated from the same probabilistic model" and "the data in c_i and c_j has two or more clusters in it". The pair of clusters that is selected for merging is the one with highest probability of the merged hypothesis.

Figure S15b shows the clusters found on all the data (on the left) as well as the consensus matrix obtained by applying BHC to 200 random subsamples of 95% of the data. This shows that, while the clustering algorithm works well on the full dataset, different clustering structures are found in the data subsamples, giving a fuzzy similarity matrix. This is due to the fact that most clusters are very small, and are hard to identify when only a subset of the data is available. The output of COCA obtained with this clustering algorithm is shown in Figure S16, the output KLIC is shown in the main paper. Higher weights are assigned on average to the expression data, with an average of 0.58.

S4.1.2 PAM with Gower's distance

Another clustering algorithm that could have been applied to this dataset is PAM with Gower's distance (Gower, 1971). In this case, all variables are binary and therefore Gower's distance is equivalent to Jaccard's distance. For two multivariate binary observations x_i and x_j, this is defined

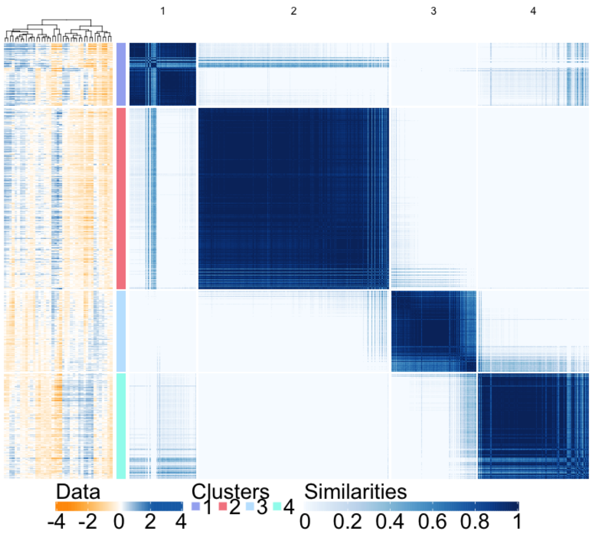

(a) Expression data, PAM.
Cophenetic correlation coefficient: 0.971.

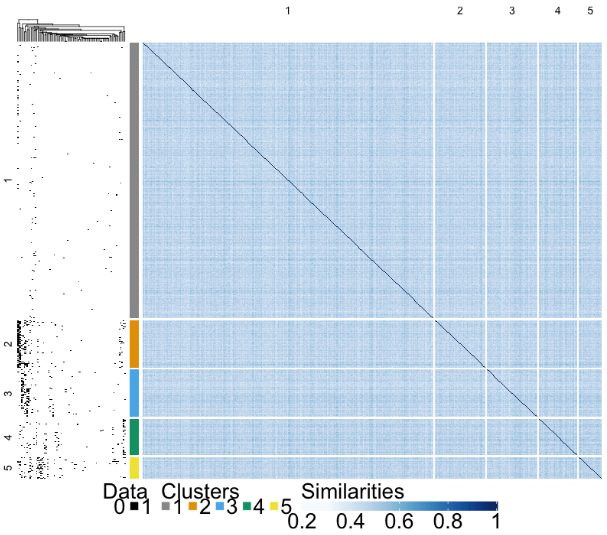

(b) ChIP data, BHC.
Cophenetic correlation coefficient: 0.103.

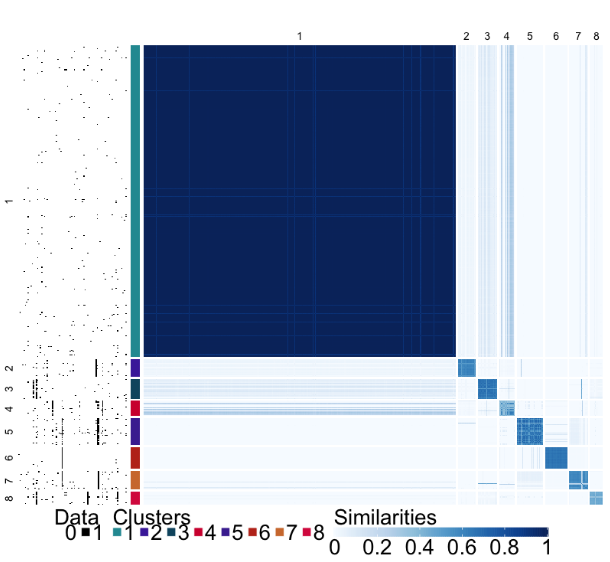

(c) ChIP data, PAM.
Cophenetic correlation coefficient: 0.996.

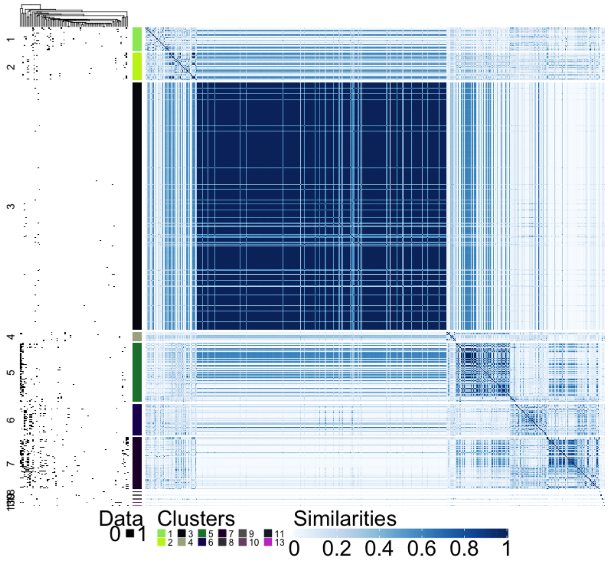

(d) ChIP data, GBNP.
Cophenetic correlation coefficient: 0.931.

Figure S15. Consensus matrices.

as one minus the Jaccard index:

$$J = \frac{M_{11}}{M_{01} + M_{01} + M_{11}}, \tag{9}$$

where M_{11} is the number of variables where x_i and x_j both have value of 1, M_{01} is the number of variables where x_i is 0 and x_j is 1 and viceversa for M_{01}. This distance is particularly suited for this dataset because here the ones correspond to transcription factors that are believed with high confidence to be able to bind to the promoter region of the corresponding gene, whereas zeros are transcription factors for which we are not able to reject the hypothesis that they do not bind to that promoter region. Thus, in a sense, ones carry more information than zeros.

The consensus matrix obtained by subsampling 200 times 95% of the data is shown in Figure S15c, the output of COCA and KLIC in Figures S16 and S17 respectively. Details on how the number of clusters was chosen are given in Section S4.2. As usual, the number of clusters for KLIC and COCA was chosen in order to maximise the silhouette. KLIC selected $K = 3$ and COCA $K = 10$. GOTO scores for the clustering found with PAM algorithm and Gower's distance, as well as those given by KLIC and COCA for three and ten clusters are reported in Table S1. Higher weights are assigned to the ChIP data, with an average of 0.78.

S4.1.3 Greedy Bayesian non-parametric clustering algorithm

The last clustering algorithm that we considered is a greedy approximation to the Gibbs sampling algorithm for Dirichlet process mixture models of Neal (2000). In the greedy version of the algorithm used here at each iteration cluster allocations are made in a deterministic fashion, assigning each observation to the cluster with highest probability, instead of sampling the cluster labels according to their conditional probabilities.

Figure S15d shows the consensus matrix, Figures S16 and S17 show the output of COCA and KLIC respectively. (Note that, for brevity, we refer to this method as "GBNP", which stands for Greedy Bayesian NonParametric algorithm.) Higher weights are assigned to the ChIP data points, with an average of 0.59.

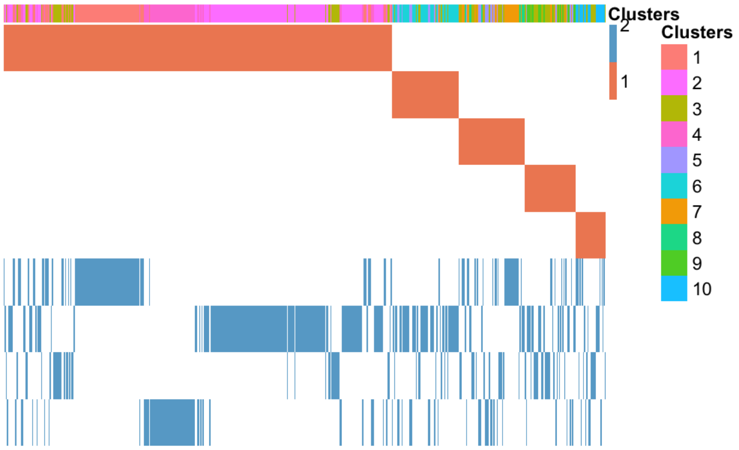

(a) BHC

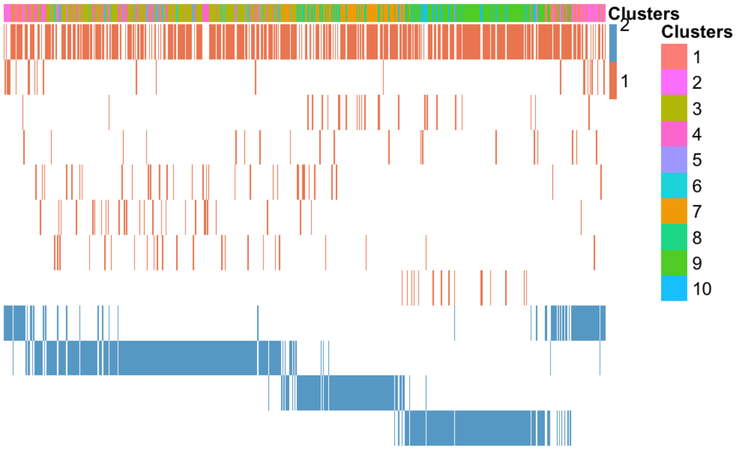

(b) PAM with Gower's distance.

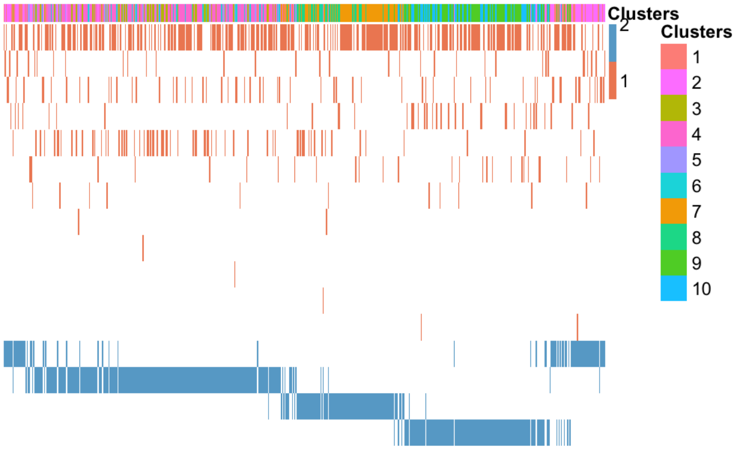

(c) GBNP.

Figure S16. Transcriptional module discovery. Output of COCA.

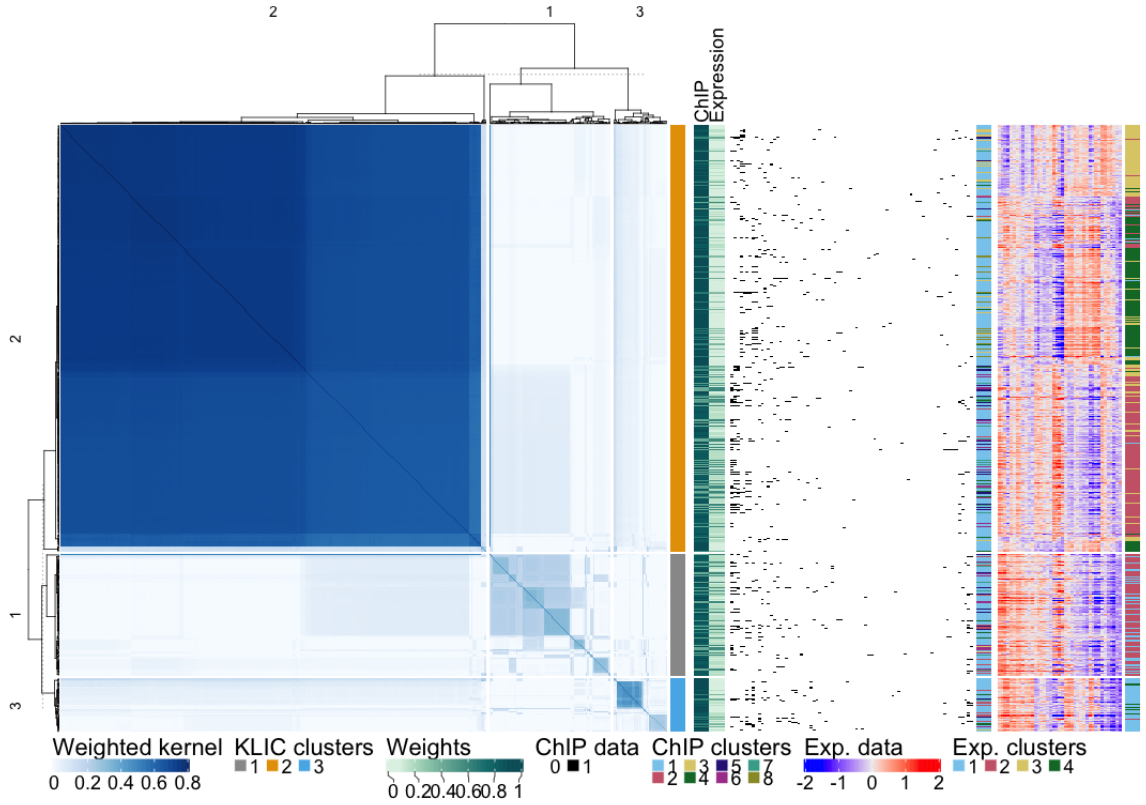

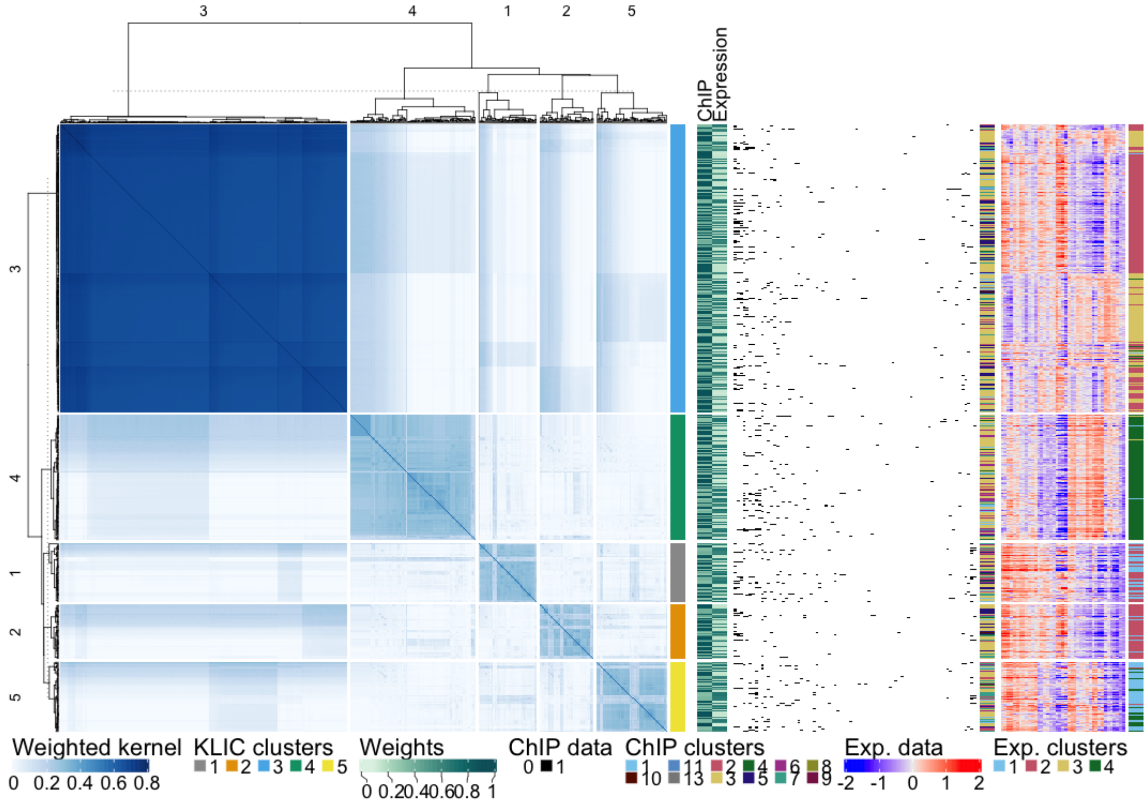

Figure S17. Transcriptional module discovery. Output of KLIC. PAM with Gower's distance (above) and GBNP (below).

Clusters	Dataset(s)	Algorithm	GOTO BP	GOTO MF	GOTO CC
4	Expression	PAM correlation	6.1194	0.9075	8.4139
8	ChIP	PAM Gower's	6.0872	0.8959	8.3261
5	ChIP	BHC	6.0020	0.9192	8.2886
12	ChIP	GBNP	6.0192	0.9176	8.3664
4	ChIP+Expression	COCA (PAM + BHC)	6.1194	0.9075	8.4139
4	ChIP+Expression	KLIC (PAM + BHC)	6.1221	0.9074	8.4103
10	ChIP+Expression	COCA (PAM + BHC)	6.2767	0.9347	8.5137
10	ChIP+Expression	KLIC (PAM + BHC)	6.3240	0.9473	8.5310
3	ChIP+Expression	COCA (PAM + PAM)	5.9609	0.8991	8.2780
3	ChIP+Expression	KLIC (PAM + PAM)	5.9188	0.8915	8.1766
10	ChIP+Expression	COCA (PAM + PAM)	6.3429	0.9211	8.5126
10	ChIP+Expression	KLIC (PAM + PAM)	6.3724	0.9094	8.4868
5	ChIP+Expression	COCA (PAM + GBNP)	6.1298	0.9078	8.4218
5	ChIP+Expression	KLIC (PAM + GBNP)	5.9629	0.9108	8.3246
10	ChIP+Expression	COCA (PAM + GBNP)	6.1605	0.9118	8.4796
10	ChIP+Expression	KLIC (PAM + GBNP)	6.2277	0.9262	8.4814

Table S1. Gene Ontology Term Overlap scores for different sets of data, clustering algorithms and numbers of clusters. “BP” stands for “biological process” ontology, “MF” for “molecular function”, and “CC” for “cellular component”.

S4.2 Choice of the number of clusters

In order to choose the number of clusters when using PAM, we considered multiple metrics: the average silhouette (Rousseeuw, 1987), the gap statistic (Tibshirani et al., 2001), and the original and modified versions of Dunn's index (Dunn, 1974, Halkidi et al., 2001). We considered all number of clusters from two to 20. These are shown in Figures S18 and S19. For the expression data, we chose four clusters since three of the chosen metrics have a peak at $K = 4$. For the ChIP data, there is no consensus among the metrics, so we selected $K = 8$ based on the gap metric.

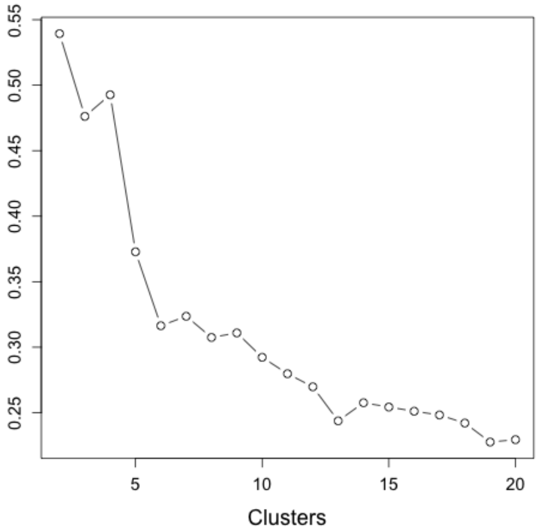

(a) Average silhouette.

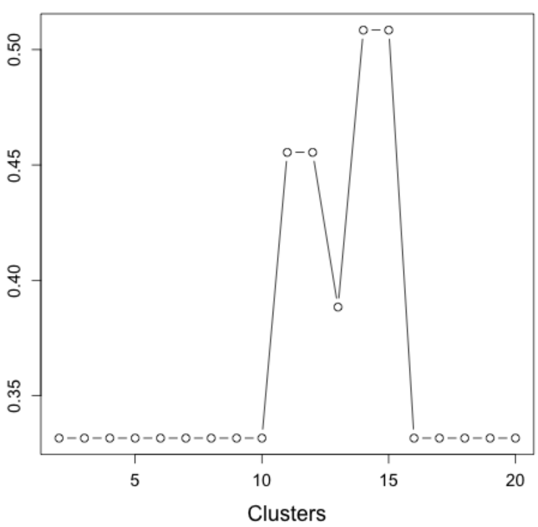

(b) Widest gap.

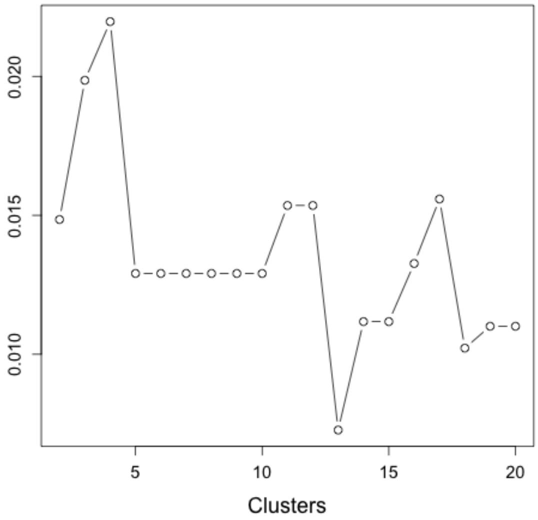

(c) Dunn's index.

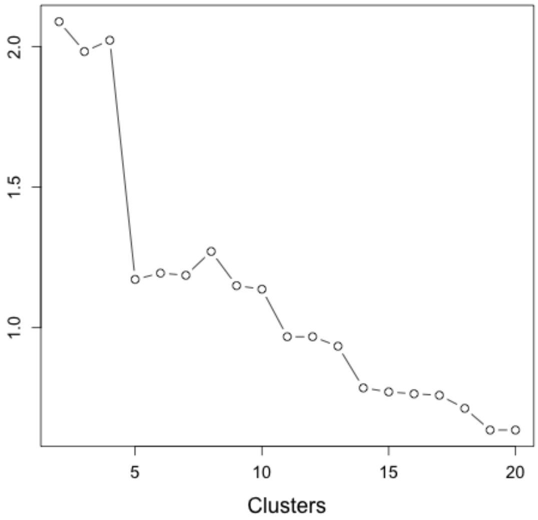

(d) Dunn's modified index.

Figure S18. Expression data. Metrics used to choose the number of clusters.

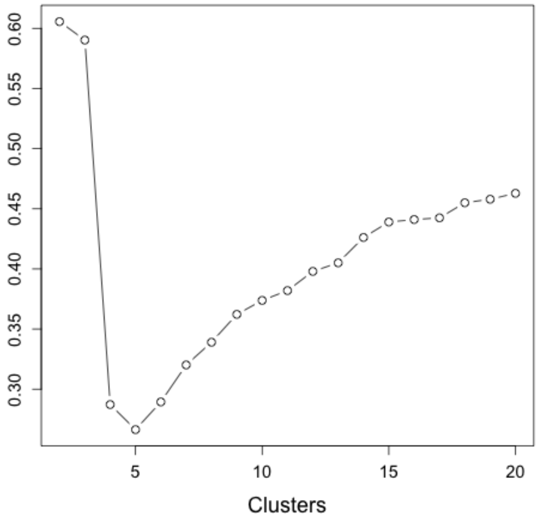

(a) Average silhouette.

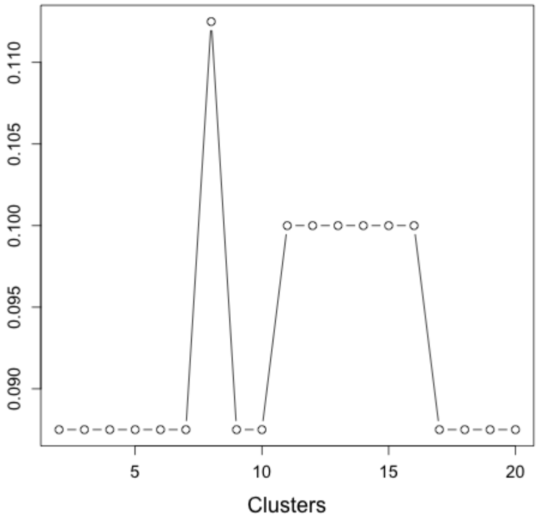

(b) Widest gap.

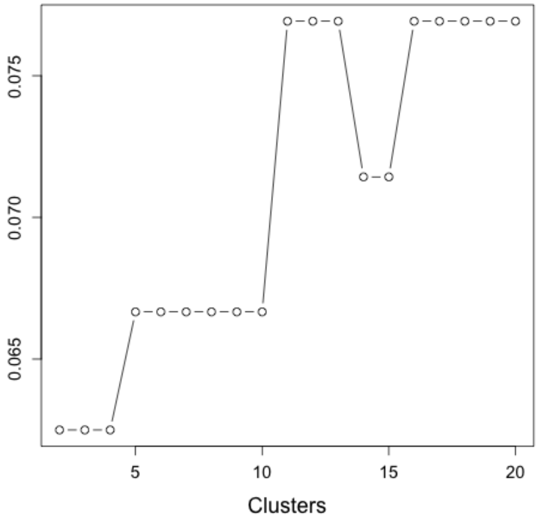

(c) Dunn's index.

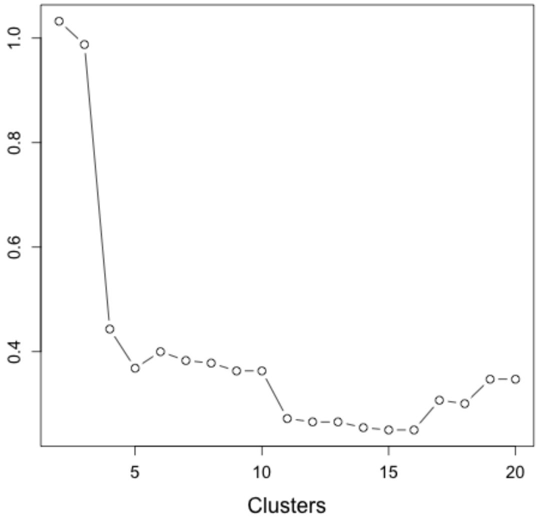

(d) Dunn's modified index.

Figure S19. ChIP data. Metrics used to choose the number of clusters.

References

Brunet, J.-P., Tamayo, P., Golub, T. R., and Mesirov, J. P. (2004). Metagenes and molecular pattern discovery using matrix factorization. *Proceedings of the national academy of sciences*, 101(12):4164–4169. *Referred to on page S8.*

De Hoon, M. J., Imoto, S., Nolan, J., and Miyano, S. (2004). Open source clustering software. *Bioinformatics*, 20(9):1453–1454. *Referred to on page S17.*

Dunn, J. C. (1974). Well-separated clusters and optimal fuzzy partitions. *Journal of cybernetics*, 4(1):95–104. *Referred to on page S26.*

Girolami, M. (2002). Mercer kernel-based clustering in feature space. *IEEE Transactions on Neural Networks*, 13(3):780–784. *Referred to on page S2.*

Gönen, M. and Margolin, A. A. (2014). Localized data fusion for kernel k-means clustering with application to cancer biology. In *Advances in Neural Information Processing Systems*, pages 1305–1313. *Referred to on page S2.*

Gower, J. C. (1971). A general coefficient of similarity and some of its properties. *Biometrics*, pages 857–871. *Referred to on page S20.*

Halkidi, M., Batistakis, Y., and Vazirgiannis, M. (2001). On clustering validation techniques. *Journal of intelligent information systems*, 17(2-3):107–145. *Referred to on page S26.*

Heller, K. A. and Ghahramani, Z. (2005). Bayesian hierarchical clustering. In *Proceedings of the 22nd international conference on Machine learning*, pages 297–304. ACM. *Referred to on page S20.*

Hoadley, K. A., Yau, C., Wolf, D. M., Cherniack, A. D., Tamborero, D., Ng, S., Leiserson, M. D., Niu, B., McLellan, M. D., Uzunangelov, V., et al. (2014). Multiplatform analysis of 12 cancer types reveals molecular classification within and across tissues of origin. *Cell*, 158(4):929–944. *Referred to on pages S1, S13, S14, and S15.*

Neal, R. M. (2000). Markov Chain Sampling Methods for Dirichlet Process Mixture Models. *Journal of Computational and Graphical Statistics*, 9(2):249–265. *Referred to on page S22.*

Rousseeuw, P. J. (1987). Silhouettes: A graphical aid to the interpretation and validation of cluster analysis. *Journal of Computational and Applied Mathematics*, 20(C):53–65. *Referred to on page S26.*

Schölkopf, B. et al. (1998). Nonlinear component analysis as a kernel eigenvalue problem. *Neural computation*, 10(5):1299–1319. *Referred to on page S3.*

Shawe-Taylor, J. and Cristianini, N. (2004). *Kernel methods for pattern analysis*. Cambridge University Press. *Referred to on page S3.*

Sokal, R. R. and Rohlf, F. J. (1962). The comparison of dendrograms by objective methods. *Taxon*, 11(2):33–40. *Referred to on page S8.*

Steinhaus, W. H. D. (1956). Sur la division des corps matériels en parties. *Bulletin de l'Académie Polonaise des Sciences*, IV(12):801–804. *Referred to on page S2.*

Tibshirani, R., Walther, G., and Hastie, T. (2001). Estimating the number of clusters in a data set via the gap statistic. *Journal of the Royal Statistical Society: Series B (Statistical Methodology)*, 63(2):411–423. *Referred to on page S26.*
